# Research Progress and Application of Polyimide-Based Nanocomposites

**DOI:** 10.3390/nano13040656

**Published:** 2023-02-08

**Authors:** Jinming Ma, Xiangfu Liu, Rongwen Wang, Chengxu Lu, Xueqin Wen, Guoli Tu

**Affiliations:** Wuhan National Laboratory for Optoelectronics, Huazhong University of Science and Technology, 1037 Luoyu Road, Wuhan 430074, China

**Keywords:** polyimide nanofiller composites, molecular design, manufacturing process, combination methods, expand applications

## Abstract

Polyimide (PI) is one of the most dominant engineering plastics with excellent thermal, mechanical, chemical stability and dielectric performance. Further improving the versatility of PIs is of great significance, broadening their application prospects. Thus, integrating functional nanofillers can finely tune the individual characteristic to a certain extent as required by the function. Integrating the two complementary benefits, PI-based composites strongly expand applications, such as aerospace, microelectronic devices, separation membranes, catalysis, and sensors. Here, from the perspective of system science, the recent studies of PI-based composites for molecular design, manufacturing process, combination methods, and the relevant applications are reviewed, more relevantly on the mechanism underlying the phenomena. Additionally, a systematic summary of the current challenges and further directions for PI nanocomposites is presented. Hence, the review will pave the way for future studies.

## 1. Introduction

Polyimides (PIs) are a special engineering plastic listed as one of the most promising engineering plastics in the 21st century. PIs are a class of polymers containing imine heterocyclic repeating units, which were first discovered in 1908 by Bogert and Renshaw [1]. The first commercial aromatic type was produced by two-step condensation polymerization in 1955, using the monomers 4,40-diaminodiphenyl ether (ODA) and pyromellitic dianhydride (PMDA) [2]. Up to now, many scholars have deeply studied PI-related materials (more than 2000 published papers yearly since 2016). PIs are divided into aromatic, semiaromatic, and aliphatic groups [3]. Aromatic PIs still keep a certain quality at a high temperature of ~ 500 °C and a boiling point of liquid nitrogen of −196 °C, which have a broader usage temperature range than other polymers [4]. They can be processed into film, foam, plastic, fiber, and other forms [5,6,7,8,9,10]. For their excellent thermal stability, superior insulation properties, high solvency, chemical corrosion resistance, and strong mechanical capacities, they are widely used in aerospace, microelectronic devices, gas separation membranes, photocatalysis, and sensors [11,12,13,14,15,16]. In addition to those mentioned above, modified PI-films can produce high-frequency flexible copper-clad laminate, which can be used in automatic driving, smart homes, 5G mobile phones, radar, and other fields [17,18,19,20].

With the constantly increasing demand for material properties and functions in various high-tech fields, single PI materials have exhibited certain deficiencies in these areas with special requirements for material properties, and their applications are severely limited. In recent years, functional materials with PIs as the main body have gradually become the research focus. Because of their excellent properties and their advantages of being used under harsh conditions, they have been selected as carrier materials. Nanoscale materials have specific surface effects, unique small-size effects, quantum-size effects, and macroscopic quantum tunneling effects. These properties strongly affect the electrical, thermal, optical, and mechanical properties. Therefore, these nanofillers with specific properties can be integrated into the PI matrix, giving their composites more particular functions and extending their application scene. Due to the unique preparation process of PIs, the reported nanofillers are integrated inside bulk PI, mainly including in situ polymerization [21,22,23], sol–gel [24,25,26], solution blending [27], electrospinning [28,29], or in combination with the PI surface, such as deposition [30,31] and surface ion exchange [32,33], to regulate the final performance of obtained composites. Inorganics, including ceramics, clay, silica, molecular sieves, and so on; metals and their oxides, such as gold (Au), silver (Ag), platinum (Pt), copper (Cu), iron (Fe), cobalt (Co), nickel (Ni), aluminum (Al), chromium (Cr), and tin (Sn); and organic materials, such as carbon nanotubes (CNT) and graphene, are usually adopted as the favorable nanofillers [34,35,36,37,38,39,40,41,42,43,44,45]. By hybridizing with the nanostructures mentioned above, the thermal, mechanical, photoelectric, solvent resistance, or gas permeability of PIs can be adjusted to achieve the target performance to a certain extent. Combining the two has complementary advantages to expand the application field significantly. Here, this review briefly summarizes the current research progress of PI-based composites, including molecular design, manufacturing process, combination methods, and their relevant applications. Hence, the study will pave the way for further research on PI-based nanocomposites.

## 2. PI Basic Information

### 2.1. PI Molecular Structure

**Figure 1:**
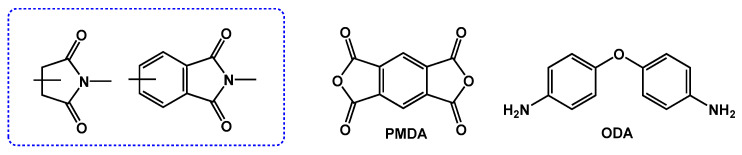
The characteristic imide rings in the PI backbone and the structure of PMDA and ODA.

PIs are one class of high-performance polymers containing imide rings (Figure 1). They exhibit superior performance driven by their rigid aromatic structures and imide rings [45,46,47,48]. Since aromatic PI was first prepared, PIs have developed into various aliphatic, aromatic, and aliphatic–aromatic structures and dominant engineering plastic for high-temperature resistance applications [49,50,51]. The most well-known commercialized PI film, Kapton™, as shown in Figure 1, was synthesized by Sroog55 with the monomers ODA and PMDA. Afterward, DuPont realized its industrialization, which owns a glass transition temperature (T_g_) reaching up to 385 °C and thermal decomposition temperatures (T_d_) over 500 °C, rendering it capable of being used for a long time at 230 °C [52]. Favorably, the first high-performance flexible organic light-emitting diode (OLED) display screen was fabricated on a PI substrate [53].

**Figure 2:**
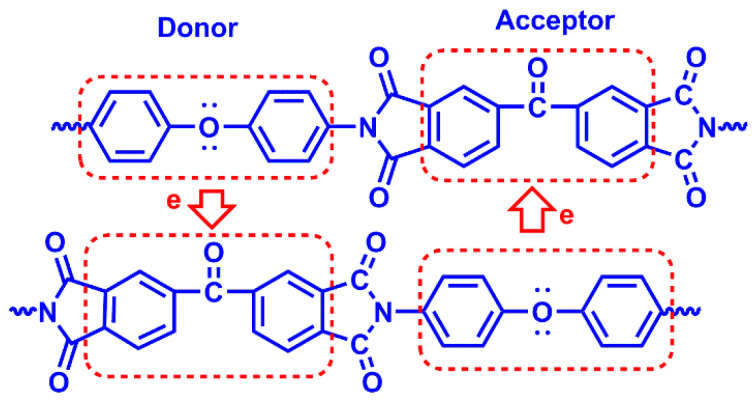
The schematic of intra- and intermolecular charge transfer complexes (CTC).

However, the color of aromatic PI films is typically yellow or brown, with poor light transmittance. For instance, Kapton™, with a thickness of 80 μm, exhibits a brown color, and its cut-off wavelength (initial wavelength corresponding to light transmittance exceeding 1%, *λ*_0_) locates as long as 455 nm. A 50 μm Kapton™ obtains light transmittance (T%) of merely 80.9% at 760 nm [54]. The deep color of PI films results from the formation of intra- and intermolecular charge transfer complexes (CTCs, as plotted in Figure 2) between aromatic dianhydrides (electron acceptors) and diamines (electron donors), causing strong filtration in the visible region. The generation of CTCs depends on the energy gap between the highest occupied molecular orbital (HOMO) of diamine and the lowest unoccupied molecular orbital (LUMO) of dianhydride monomers [55,56]. The larger energy gap leads to the light absorption of a shorter wavelength, a lighter color of PIs. Moreover, electron conjugation in PIs is mainly determined by molecular structure and chain aggregation state, which is essential for evaluating their transparency properties, including T%, *λ*_0_, and yellowness index (YI).

### 2.2. The Colorless PI

**Figure 3:**
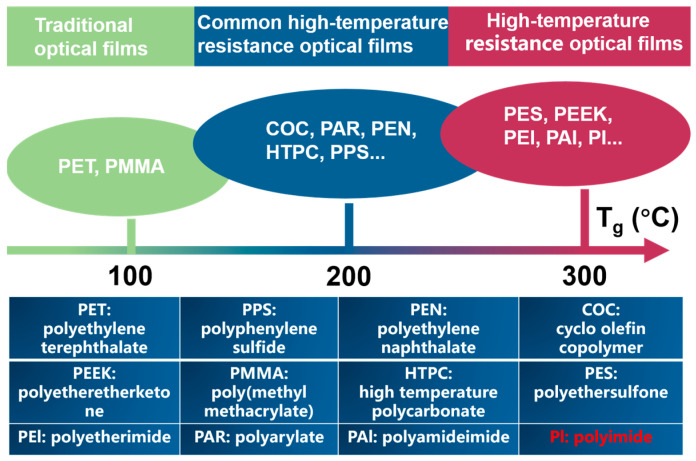
Classification of polymer optical films.

For decades, high thermal temperature resistance has been an ever-increasing demand in the rapid development of optoelectronic devices with high integration, reliability, and signal transmission speed [57]. For example, in preparing a flexible active matrix OLED (AMOLED) display screen, the processing temperature on the flexible supports might exceed 350 °C [58]. As plotted in Figure 3, the majority of common flexible polymer substrates, such as polyethylene naphthalate (PEN) with T_g_ of 123 °C, polyethylene terephthalate (PET) with T_g_ of 78 °C, or polycarbonate (PC) with T_g_ of 145 °C, own outstanding optical transparency but will be deteriorated at such a high processing temperature due to their limited service thermal stability. Thus, optically transparent substrates with high-temperature-resistant properties have attracted much attention from academic and engineering applications. Correspondingly, colorless polyimide (CPI) leads the critical position in advanced optical polymer films, considering their comprehensive performance and potential market volume [59].

Available commercial colorless polymer films with relatively high-temperature resistance properties are briefly summarized in Table 1. CPIs occupy more than 90% of the consumption market of optical polymer films.

The molecular design with controllable polymerization is one effective method to achieve CPI films with high-temperature resistance and simultaneous excellent optimal transparency. Meanwhile, the fine-tuning aggregation state of the PI chain during film preparation and post-treatment is also critical. The main principle for a CPI structural design is to weaken the CTC effect in PI, namely, reduce the electron-donating ability in diamine and the electron-accepting ability in dianhydride via the introduction of different functional groups or monomers. For the molecular design of CPIs, the balance among their thermal resistance, optical transparency, mechanical/dielectric characteristics, and other properties is challenging. This usually accounts for these targets contradicting each other in most cases. For instance, managing to improve the optical transparency of CPI films, the insertion of alicyclic and highly twisted substituents inevitably hinders their thermal stability and vice versa.

**Figure 4:**
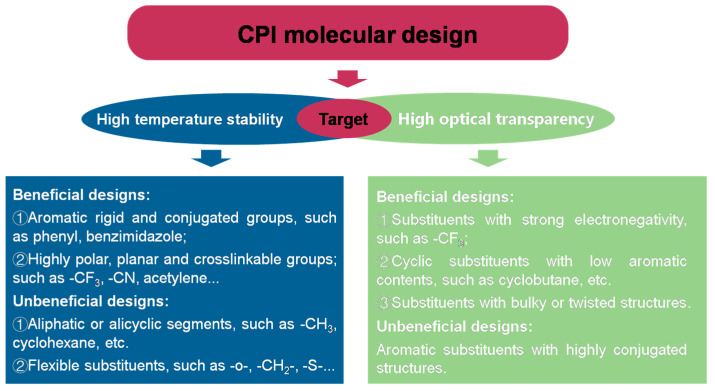
Schematic of CPI molecular design principles. Reprinted with permission from Ref. [61]. 2015, Elsevier.

Figure 4 summarizes the design principles of CPI molecular, including favorable and unfavorable, designs for developing high-performance CPIs. The favorable steps could enhance both the optical transparency and high-temperature resistance for CPI, involving the introduction of strongly electronegative groups (e.g., trifluoromethyl group), alicyclic substituents (e.g., cyclohexane and cardo groups), and asymmetrical or twisted rigid groups (e.g., asymmetrically substituted biphenyl). All these substituted structures have been extensively employed to develop new CPIs. The majority of reported CPIs are consistent with these structural features. The reported CPIs in Table 2 comprise either alicyclic structures (cyclohexane) or fluoro-containing groups (–CF_3_). These structures render CPIs possessing excellent temperature resistance (T_g_ ≥ 300 °C) and superior visible optical transparency (>85%). All the groups or structures mentioned above were utilized to prohibit the CTC effects in CPIs, which is the main reason for the deep color in PIs.

Adjusting the molecular packing density and PI chain spacing can effectively prevent the formation of CTCs and thereby enhance optical transparency. At the same time, increasing chain spacing will seriously deteriorate its temperature resistance. In recent decades, the primary strategy to enhance the CPI thermal stability is to bring some rigid and linear groups into the chains, for instance, the introduction of rigid and linear substituents with alicyclic rings and copolymerization and so on. Nevertheless, these procedures would inevitably weaken the optical properties to some extent. Thus, establishing a balance between PI’s thermal and optical capability to achieve high-performance CPI films remains challenging. Recently, our group developed a novel rigid semi-alicyclic dianhydride 8FDA, namely, (5,11-difluoro-5,11-bis (trifluoromethyl) -5,11 -dihydro- 1 H, 3H-anthraceno [2,3- c: 6, 7-c’] difuran 1,3,7,9-tetraone), which was originated from the classical monomers1S,2R,4S,5R-cyclohexanetetracarboxylic dianhydride (HPMDA) and 4,4′-(hexafluoroisopropylidene) diphthalic anhydride (6FDA). These unique 8FDA-based structures endow the resulting CPI films an obviously higher T_g_ up to 401 °C, as well as a lower coefficient of thermal expansion (CTE) of 14 ppm K^−1^, indicating that an 8FDA-based building block is a promising structural candidate for high-performance CPIs in flexible transparent OLED display application [74].

### 2.3. PI Preparation

**Figure 5:**
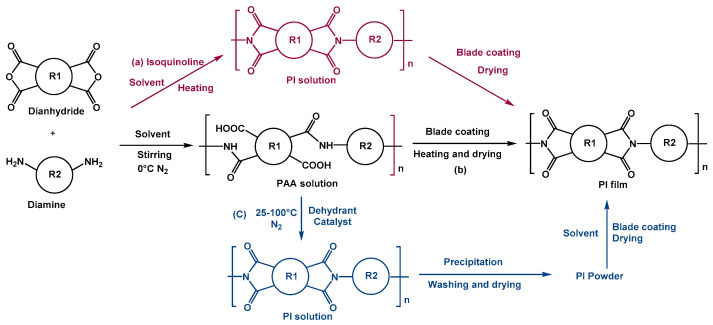
Scheme of PI imidization: (**a**) direct thermal, (**b**) two-step thermal, and (**c**) chemical imidization.

High-molecular-weight aromatic PIs with pyromellitic dianhydride and diamines retained their properties and performance up to ~500 °C and were successfully synthesized by a two-stage polycondensation in 1955. Figure 5 illustrates the simple routes for the traditional preparation of PI via the poly (amic acid) (PAA) method. The reaction of dianhydrides and diamines subsequently formed PAA and then converted to PI through thermal/chemical imidization. Typically, most high-performance aromatic PIs adopt the PAA imidization route, ascribing to their being poorly soluble in common solvents [75,76]. Dianhydride and diamine require purification, such as sublimation, to achieve stoichiometric balance, thus obtaining the high viscosity of the PAA solution. Note that dianhydrides are very sensitive to water, and diamines are easy to be oxidized by oxygen. Hence, the reactant solvent should be desiccated, and the polymerization must be conducted in oxygen-free conditions (in Ar or N_2_) [77]. Finally, the PAA is converted into high-performance PI films after imidization.

#### 2.3.1. Imidization Methods

Imidization is an essential step for successfully forming PI, including thermal and chemical imidization. Thermal imidization methods can be divided into direct thermal imidization and two-step imidization steps.

- Direct thermal imidization

Direct thermal imidization is the reaction between diamine and dianhydride, which is appropriate for the alicyclic diamine that polymerizes into insoluble salt and aliphatic dianhydride with weak reactivity (Figure 5a). Direct thermal imidization consists of high-temperature melting, solid-phase, and solution synthesis. Among them, the melting procedure only applies to the meltable PI formation; the solid-phase process is a rapid reaction step of an amide salt monomer without rigorous moisture control; the solution synthesis procedure mainly uses a one-pot synthesis step. Typically, the diamine dianhydride is polymerized and imidized in a high-boiling-point solvent (e.g., NMP) with a catalyst (e.g., isoquinoline or benzoic acid), thus obtaining PI solution with a high molecular weight.

- Two-step method

The two-step synthesis method is the most commonly used (Figure 5b) of the three ways. Typically, PAA is aforehand obtained by dianhydride and diamine polymerization. Next, the PAA solution is cast on a support substrate (glass or plate) and then programmed to be heated to remove the solvent. The imidization processes are finally completed at a temperature higher than 300 °C. For instance, the PMDA-ODA system imidization occurs at 360–400 °C but only carried out in a shorter time. Some research exhibited that low imidization temperature improves the transparency of obtained PIs while impairing their thermal and mechanical performance. Generally, a temperature above T_g_ is required for complete imidization [78]. The molecular chains start to move at a temperature above T_g_, benefiting from increasing the packing density and obtaining higher T_g_ [79,80,81]. The molecular motion facilitates chains’ disorder, leading to higher CTE and poorer transparency [82,83]. Thermal imidization is usually conducted in an oven under a constant N_2_ flow to remove the byproduct of water.

- Chemical imidization

Comparatively, as shown in Figure 5c, chemical imidization demands the utilization of catalysts such as pyridine/isoquinoline and a dehydrant such as acetic anhydride. Typically, prepare the PAA solutions first. Then, add the catalyst and dehydrant into the PAA solution, subsequently achieving the PI solution after dehydration cyclization under a specific temperature. Finally, the raw product is precipitated by the poor solvent (e.g., methanol/water). After the washing, filtering, and drying processes, the solid PI product was obtained. However, the chemical imidization method requires additional heating to complete the full imidization [84]. Consequently, this method can improve obtained PIs’ transmittance due to the lower heat treatment temperature below T_g_, which benefits the avoidance of oxidation, embrittlement, and crosslinking at high temperature, thus reducing CTE and packing density [85]. Additionally, the chemical imidization process can easily close the unstable terminal amino group with the help of acetic anhydride and so on, consequently avoiding oxidation and color change at high temperature, leading to higher visible transparency and resistance.

#### 2.3.2. PI Film Preparation Techniques

Similarly, the manufacturing techniques for PI films have coincided with the common polymer films. However, the uniqueness of PI films is mainly ascribed to their relatively higher T_g_ and lower solubility in common solvents in contrast to other polymers. The precursor PAA-casting method is frequently used, which can be divided into two approaches: slice-to-slice in the laboratory and roll-to-roll in engineering.

- Slice-to-slice scale preparation of PI films in laboratory

**Figure 6:**
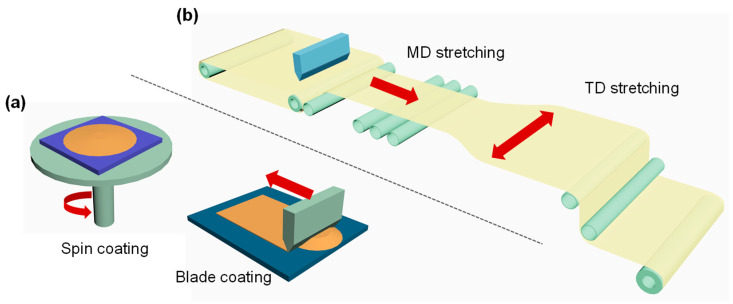
(**a**) Slice-to-slice scale (spin/blade coating) and (**b**) roll-to-roll scale (stretching process) manufacturing for PI films via PAA precursors/PI resin solution.

The commonly prepared strategies for PI films included the standard PAA route and the soluble PI resin method. In the standard PAA route, the diamine and dianhydride monomers polymerized into PAA solution in DMAc or NMP. The used PAA for PI films is generally newly synthesized due to its sensitivity to heat and moisture, thus easily degrading during longtime storage. The PAA solution is slice-to-slice cast (spin or blade-coating method) to clean supports, such as glass or plate (Figure 6a), then transferred to the oven for programmed heating for thermal imidization or cyclization, accompanied by the physical evaporation of a solvent and the byproduct elimination of water. A cyclization temperature as high as 300–350 °C is essential to complete the PI transition from PAA. Unfavorably, such a high process temperature inevitably affects the visible transparency of obtained PI films, along with defects, such as pinholes, bubbles, and cracks, during the high-temperature system. Contrastively, the utilization of PI resins in solvents, such as DMAc, can achieve PI films at a relatively low temperature because the curing procedure for the pre-imidized PI solution is nearly pure physical evaporation of the solvent (below 300 °C), thus obtaining high film transmittance and good surface smoothness. It is well established that the solubility of PI resins during this route is vital, particularly related to their molecular structures. Therefore, flexible groups such as –O– and unconjugated structures such as alicyclic groups are incorporated to facilitate the solubility increase in PI resins, which hinders the thermal and mechanical capacity of obtained PI films to a certain extent.

- Industrial roll-to-roll scale preparation of PI films

The biggest distinction in industrial-scale PI film manufacturing (roll-to-roll) is the stretching process compared with the slice-to-slice preparation [86]. In roll-to-roll, either uniaxial stretching (machine direction, MD) or biaxial stretching (transverse direction, TD) of precursor PAA films in Figure 6b will render the full orientation and extension of PI chains, thus strongly improving the mechanical performance of the obtained PI films in consideration of polymer physics. As a result, the elongations at the break of roll-to-roll PI can be extended several times, while the value of lab-making PI films is usually below 20% without any stretching treatment. Meanwhile, the roll-to-roll stretching of PI resins can effectively enhance the high-temperature optical and dimensional resistance of obtained PI films below T_g_.

In roll-to-roll, the obtained PAA solution is cast to a heated rotating stainless-steel drum to form a continuous film. The partially evaporated solvent and imidization reaction occur at the same time to obtain a gel-like PAA film. Then, the PAA film’s first MD stretches in the metal drum and regulates the stretching rate by controlling the drive source and speed using nip rolls. Then the gel film is TD stretched under the tenter clips’ outward movement, along with the solvent evaporation and biaxial-oriented PI (BOPI) film formed after heating. Such roll-to-roll routes are extensively applied for PI film production, and significant patent activity has existed since PI commercialization appeared in the 1960s. So far, the majority of available commercial aromatic PI films are produced by this procedure. Moreover, the reported CPI films derived from the copolymers of fluoro-containing dianhydride (6FDA or BPDA) and diamine (TFMB) are produced via chemical imidization of the PAAs to soluble PI resins and then obtained the tough PI films with low color and high transparency from this roll-to-roll procedure by the DuPont Company and Kolon Industries [87].

#### 2.3.3. PI Nanofiber Preparation

Polymer-based nanofibers are the focus and frontier in the field of fiber materials [88,89,90]. The size effect and surface effect brought about by fiber diameter refinement endow many unique properties [91,92]. The preparation methods of PI nanofibers include electrostatic spinning [93], centrifugal jet spinning [94,95], sonochemistry [96], solution blow spinning, and melt spinning [97,98]. Among them, electrostatic spinning is the most commonly used method because of its simple manufacturing equipment and high process controllability [99]. High-performance PI fibers with variable structures and adjustable performance can be used for high-temperature filter materials, bulletproof military clothing, aerospace parts, and so on [100,101,102].

**Figure 7:**
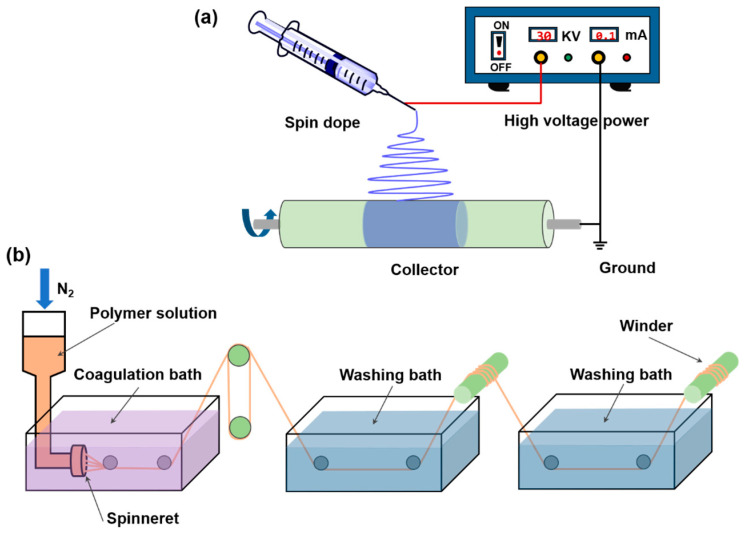
Schematic illustration of a (**a**) laboratory setup for electrospinning and (**b**) wet-spinning process.

Figure 7a shows a schematic illustration of a laboratory setup for electrospinning. High-voltage power supply, a solution container with a spinneret, and a grounded metal collector are three essential components for electrospinning. The positive electrode of the power supply is linked to the spinneret, and the negative is linked to the grounded metal collector. When the electric field force is too low to make the charged part of the solution eject, the spherical droplet suspended at the spinneret nozzle is stretched to become the charged cone. As the voltage increases beyond the critical value, the charged polymer droplets overcome the surface tension to form a jet trickle, vibrate, and whip in the air. The fiber splits, forming finer fibers that settle on the receiving device. In 1996, Chun and Reneker reported more than 20 species of polymer nanofibers, including PI, which were produced by the electrospinning technique [103]. However, in the following several years, there has been no research on electrospun PI nanofibers in the literature. Because PIs were insoluble in ordinary solvents and infusible, they were not accessible for electrospinning directly. Starting in 2003, more and more groups have been actively reporting the preparation and application of electrospun PI nanofibers, including synthesizing new types of diamine and dianhydride monomers, preparing PI nanofibers with high strength, and diverse applications [104,105,106]. Because they are generally insoluble, electrospun PI nanofibers are usually prepared by a two-step method [107]. First, PAA fiber was prepared by electrospun technology to synthesize PAA solution, and then the obtained PAA fiber was converted into PI fiber by thermal imidization.

In 2006, the team first reported nonoriented electrospun BPDA-PDA PI nanofiber membranes with a tensile strength of 210 MPa and a modulus of 2.5 GPa [108]. Then, a high-speed rotating flywheel prepared a BPD-PDA PI nanofiber strip with about 80% orientation. The average diameter of the nanofiber strip was about 180 nm, and the tensile strength of the nanofiber was up to 663.7 MPa when the imide temperature was 430 °C. Further, their group reported that the tensile strength of a copolymerized polyimide (co-PI) BPDA-BPA-ODA nanofiber strip was as high as (1.1 ± 0.1) GPa through the copolymerization method in 2008. Such high-strength polyimide nanofibers are ascribed to the highly oriented molecular chain in the ultrafine diameter range and the thermally induced orientation factors. The single fiber tensile test showed that the tensile strength of a single BPDA-PDA polyimide nanofiber with a diameter of 300 nm reached 1.7 ± 0.2 GPa. In addition, in view of the excellent heat resistance of PI, it has been found that the tensile strength of their prepared PI nanofibers can remain above 80% when the temperature is up to 350 °C [16]. High-performance PI nanofibers have been widely used in different fields.

Wet spinning is another frequently used method for Pl fiber preparation. As plotted in Figure 7b, it means to spray a spinning solvent made of fibrous polymer dissolved in a solvent from the spinneret to form a filamentary flow, and then form fibers in a coagulant bath through solvent diffusion in the filamentary flow and the penetration of the coagulant into the filamentary flow. PI fibers prepared by wet spinning can be divided into one-step (directly obtained from the spinning solution of soluble PI) and two-step (first spun to PAA precursor fibers, then obtained by subsequent imidization) methods. It is noted that the defects are usually generated by moisture volatilization in the two-step process, which will impair the final performance of the fibers. Therefore, it is necessary to manufacture the as-spun fiber with a dense structure [3,101].

Melt spinning is fabricated by extruding and stretching polymer melt. However, due to the low solubility and infusibility of most Pls, a variety of flexible units have been introduced into PI chains that endow them with good spinnability. To melt Pl, the melt-spinning temperature of Pl fibers is always too high, and the melt extrusion of PI powders would eliminate the need for solutions, coagulating baths, and additional processing to remove volatiles and achieve thermal imidization. In recent years, with the development of synthesis and spinning technologies, LaRC, R-BAPB, and ULTEM are successfully prepared by melt extrusion.

In the process of crystallizing spinning, the liquid-crystal region of the anisotropic solution or melt is easy to orient under shear and tensile flow, and the phase transformation of the anisotropic polymer will occur during the cooling process to form a high crystalline solid so that the high-strength fiber with high orientation and high crystallinity can be obtained. Few reports about the preparation of Pl fibers by liquid crystal spinning have been reported due to the difficulty of forming soluble PAAs’ liquid-crystal phase.

## 3. PI-Based Composites and Their Preparation Method

With the industry developing, materials owning special properties are mainly required in certain aspects. The application of single materials is relatively limited. To meet multiple fields’ demands, composites combining the advantages of functional organic/inorganic components to compensate for the shortage of a single characteristic are preferred. In recent decades, considerable attention has been devoted to building PI-based composites. Many preparation methods for PI composites were proposed. The nanofillers are integrated inside bulk PI, mainly including in situ polymerization, sol–gel, solution blendings, electrospinning, or in combination with the PI surface, such as deposition and surface ion exchange.

### 3.1. In Situ Polymerization

**Figure 8:**
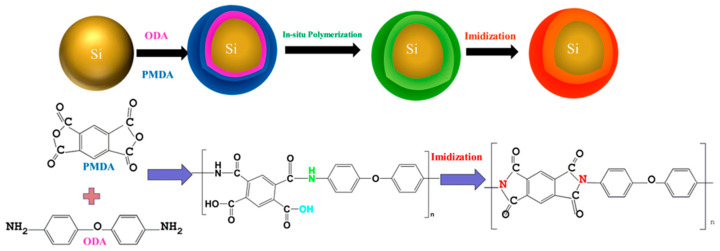
Schematic illustration of the synthesis procedure of Si@PI composites. Reprinted with permission from Ref. [111]. 2022, Wiley.

In situ polymerization is the uniform dispersion of functional nanomaterials in the PI monomers and polymerization along with PAA to PI under certain conditions, such as “improved welding”; the active site in PAA is transferred to target nanomaterials by grafting, and then heat imidizating, consequently in situ achieving the composites. Through in situ polymerization, researchers prepared a variety of PI composites, such as boron nitride/PI (BN/PI), graphene/PI, and boron carbide/PI (B_4_C/PI) composite films [109,110]. Li et al. [111] realized microencapsulation technology through in situ polymerization and imidization of PI shells on the silicon surface, which effectively alleviated the volume change of nanosilicon in the alloy/dealloying reaction and helped to obtain a flexible, uniform solid electrolyte film with Li^+^ conductivity on the electrode surface (Figure 8). This method is suitable for large-scale production, making the application of this silicon matrix composite in lithium-ion batteries promising [112,113].

**Figure 9:**
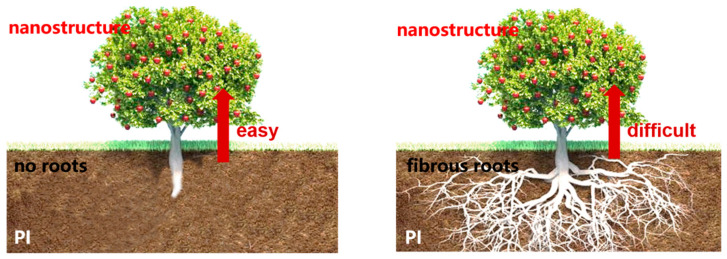
Schematic diagram of strong adhesion of in situ methods.

Chen et al. [114] prepared multiple-scale carbon fiber–carbon nanotube PI composites (CF–CNT/PI) by ultrasonic dispersion in situ polymerization. These results exhibited that the friction coefficient and wear rate of CF–CNT/PI composites were reduced by 22% and 72%, respectively, compared with pure PI, which obviously improved PI’s friction and wear performance. CF–CNT prepared by this method achieved good interfacial adhesion with the PI matrix and became an excellent self-lubricating material. As shown in Figure 9, the grafting CNT, like the fibrous roots in the tree, spreading into the soil, greatly enhances the interaction between the main root and soil, making the tree difficult to draw out from the soil. While the no-roots tree is easily pulled out due to the poor combination. It is worth noting that through the analysis and testing of the synthesized PI composite properties, the composited characteristics of high-temperature resistance, good wear resistance and self-lubrication, and excellent insulation performance were observed (Figure 9).

### 3.2. Solution Blending

**Figure 10:**
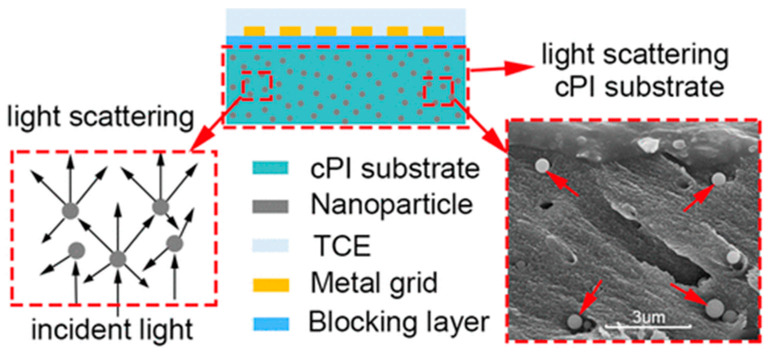
Schematic diagram of scattering layer and metal grid in PI substrate. Reprinted with permission from Ref. [116]. 2017, ACS Publications.

Solution blending is a method of dispersing nanomaterials into the corresponding solvent (DMF, PAA) of PAA and adding to the PAA solution or directly dispersing nanomaterials into the PAA solution, then obtaining PI composites after thermal imidization. Kwon et al. [115] synthesized carbon black/PI (CB/PI) composites by this solution blending. The T_d_ of CB-PI was increased by 76 °C, the T_g_ was increased by 204 °C, and the mechanical strength was increased by 16% compared with pure PI, showing superior thermal and mechanical properties. Our group attempted dispersed silica (SiO_2_) or alumina (Al_2_O_3_) nanoparticles in PAA solution to prepare SiO_2_/PI and Al_2_O_3_/PI composite films, doped with the same mass fraction of 3%, which significantly increased the haze of the two films when the light transmittance was basically unchanged or slightly reduced (Figure 10). This transparent composite PI with high haze and acceptable light transmittance is ideal for large-area flexible OLED lighting panel substrates with scattering phenomena [116]. Research about highly porous silica/PI (SiO_2_/PI), dimethicone/PI, three-phase PI/graphene/barium titanate composites, and so on have been reported using this method [117,118,119,120]. The prepared composites have the strengths of easy processing, large porosity, superior thermal stability, and high thermal degradation temperature.

### 3.3. The Deposition Method

**Figure 11:**
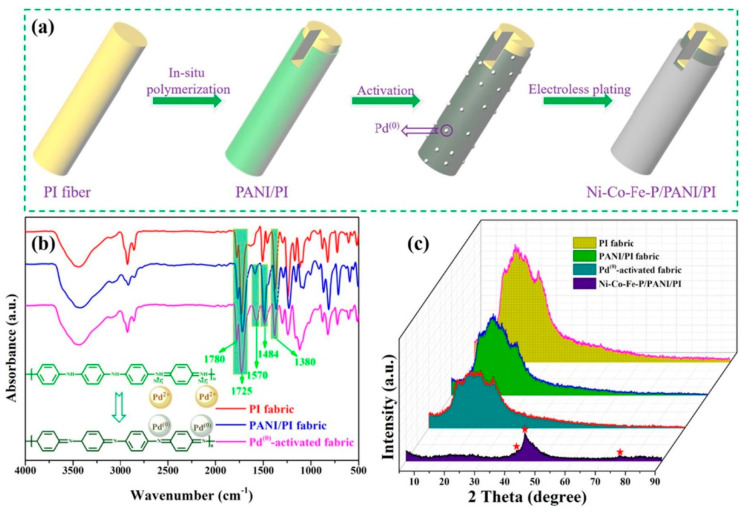
(**a**) Schematic diagram for the procedure of the Ni–Co–Fe–P/PANI/PI fabric. (**b**) FTIR spectra of PI, PANI/PI, and Pd^(0)^-activated PANI/PI. Insert image shows the reduction process from Pd^(II)^ to Pd^(0)^. (**c**) XRD patterns of PI, PANI/PI, Pd^(0)^-activated PANI/PI, and Ni–Co–Fe–P/PANI/PI.Reprinted with permission from Ref. [125]. 2020, Elsevier.

The deposition method uses physical deposition, chemical deposition, electroless coating, and other techniques to deposit nanostructures on the surface of PI and obtain the composite materials [121,122,123,124]. Wang et al. [125] used the electroless coating process to prepare blended fabrics with a multilayer structure of nickel–cobalt–ferrum–phosphorus/polyaniline/PI(Ni-Co-Fe-P/PANI/PI), which prepared high-efficiency electromagnetic shielding materials with dense metal layers, low reflection, and strong absorption characteristics, especially meeting the requirements of high temperature, high pressure, or foldable systems (Figure 11). Ishida et al. [126] prepared to shape memory alloy Ti–Ni–Cu thin films on heated PI substrates by the sputter-deposited technique. The composite film has a promising application as a small and convenient driver. Magnetic materials, metals, alloys, and semiconductors can be deposited on the PI substrate surface used in the same way, and the thickness and purity can be adjusted according to the process conditions [127,128].

Before functional layer deposition, UV/O_3_ treatment is usually used to modify the surface of various substrates in the manufacturing process of semiconductor silicon wafers and microelectronic devices. O_3_ treatment can achieve high-energy UV radiation without vacuum, and is an effective method to improve the surface wettability of PI films. It can form continuous images and texts of the required width on the film surface.

### 3.4. Electrospinning

**Figure 12:**
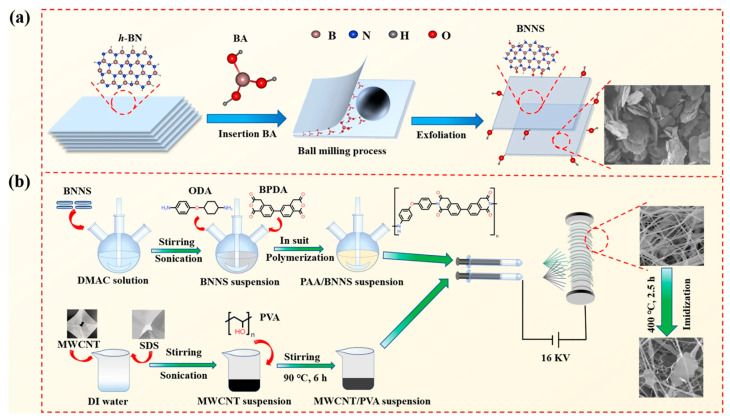
Schematic illustration for the (**a**) exfoliation of BNNS and (**b**) fabrication of PI/BNNS/CNT@αPVA membranes. Reprinted with permission from Ref. [93]. 2021, Springer.

As mentioned above, electrospinning is a process in which electrically conductive droplets are drawn and stretched by electric field force generated by a high-voltage electric field to form nanofibers. Specifically, PI composite nanofibers were developed by adding organic/inorganic nanoparticles to PI or PAA precursors and adopting suitable fiber formation processes. Zhang et al. constructed an interpenetrating carbon nanotube@carbonized polyvinyl alcohol (CNT@αPVA) network by co-electrospinning PAA/boron nitride nanosheets’ (PAA/BNNS) precursor fibers and PVA/CNT precursor fibers and high-temperature treatment (Figure 12). The membrane has a sufficiently high volume resistivity of 1015 Ω·cm and acid and alkali resistance and is self-extinguishing, which provides an effective method for developing continuous heat conductive networks in PI-based thermal management materials.

Hou et al. synthesized a PI/TiO_2_ composite by electrospinning, and the introduction of Ti changed the original PI network structure, destroyed the initial spatial arrangement of its organic polymer, and formed a new fibrous structure, with up to 90% of rhodamine B (RhB) degradation ability. PI-based composites’ nanofibers prepared by electrospinning technology achieve remarkable mechanical properties and controllable material structure, widely used in critical technical fields, such as microelectronic devices, sensors, membranes, and tissue scaffolds [129,130,131].

### 3.5. Sol–Gel Method

**Figure 13:**
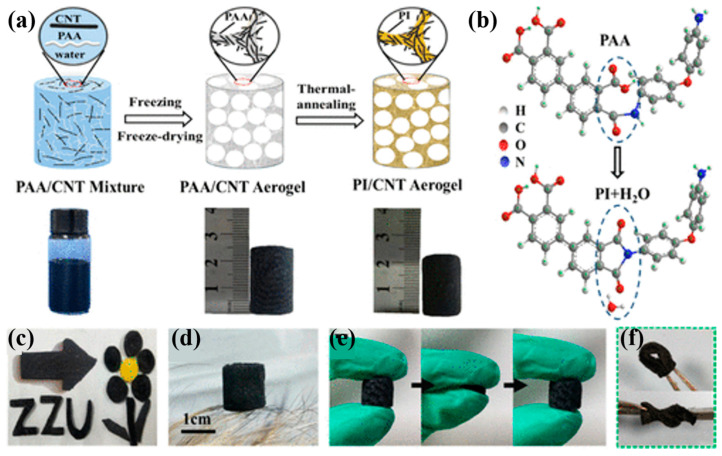
Schematic illustration of the (**a**) process of PI/CNT composite aerogel and (**b**) the thermal imidization process of PAA. Optical photograph of the (**c**) PI/CNT composites and (**d**) PI/CNT composites supported by wool. The composites withstand a high-level (**e**) compression deformation and (**f**) bending and twisting. Reprinted with permission from Ref. [134]. 2019, ACS Publications.

The sol–gel method is another reported way to make PI composite materials. Typically, cosolvent metal alkyl oxides or nanofillers with PAA or PI monomers were formed, hydrolyzed condensation to achieve a transparent sol system, and slowly polymerized between colloids to form a gel with a three-dimensional network structure. It was finally heated to volatilize small molecules such as solvent and water to obtain PI composites [132,133]. Chen et al. [134] proposed a simple freeze-drying technology to synthesize PI/CNT composite aerogels lightly and compressibly. The strong chemical interaction between the two interfaces renders hybrid aerogels that can withstand up to 80% compression without plastic deformation during 180° bending and 360° torsion (Figure 13). This proves its potential application value in high-performance wearable pressure sensors. Lin et al. [135] took advantage of the residual amino group in PI to organic–inorganic bonding with the coupling agent propyl triethoxysilane isocyanate (ICTOS) and changed the tetraethoxysilane (TEOS) amount to control a silicon content of 5–15% in the composite film. As a result, the mechanical properties of the obtained PI-polysiloxane (PI-PSA) composite material are significantly improved, and the tensile strength can reach up to 105.4 MPa when the mass fraction of nanosilica is 10%. Among them, PI-PSA hybrid materials, SiO_2_@GO hybrid modified PI composites, and so on are successfully synthesized using a similar method. By contrast, this sol–gel method possesses certain advantages. For example, it can achieve uniform doping at the molecular level, superior ultraviolet shielding performance in the ultraviolet region, good light transmittance in the visible region, and excellent thermal stability.

### 3.6. Surface Ion Exchange Method

The surface ion exchange method utilizes a specific concentration of alkali to surface-cleave the prepared PI, resulting in abundant carboxylate in the backbone, then incorporating target cation (such as Ag and Cu) in PI chains by ion exchange reaction, finally generating PI–metal composites after metallic reduction by reductant or high-temperature treatment. Nawafune et al. [32] reported that copper ions are initially doped with carboxylate anions in the hydrolyzed PI layers through surface ion exchange and then chemically reduced by a good reducing agent (dimethylamine borane). This reduction allows the diffusion of copper ions toward the film surface to form thin copper films. It simultaneously controls the fabrication of interfacial microstructures between the copper and underlying PI.

**Figure 14:**
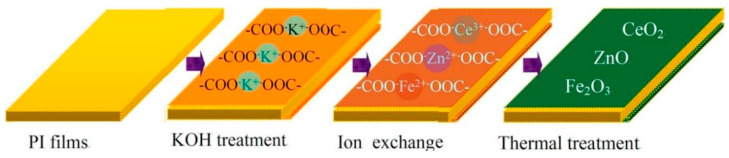
Schematic process of the PI-based CeO_2_–Fe_2_O_3_–ZnO mixed oxide preparation. Reprinted with permission from Ref. [136]. 2018, Elsevier.

Lei et al. [136] successfully prepared CeO_2_–Fe_2_O_3_–ZnO compound oxide layers on a PI substrate by this ion exchange scheme and studied the effects of different initial Ce, Fe, and Zn ion loadings on the microstructure, thermal properties, and catalytic performance of the obtained PI-supported mixed oxide, consequently achieving a superior methyl orange degradation rate of 98.7% in 12 min (Figure 14). Researchers effectively synthesized a series of PI composites using an ion exchange method, such as silver/PI (Ag/PI), copper/PI (Cu/PI), zinc oxide/PI (ZnO/PI), iron/PI (Fe/PI) composites, and so on [137,138,139,140]. The main advantage of PI composites prepared by this technology is the well dispersibility, size adjusting, and morphology modification of metal and metal oxide nanoparticles to optimize the performance. Moreover, this method can easily control the distribution of mixed metal ions in the horizontal and vertical PI matrix, thus obtaining heterostructured nanostructures on the PI surface. At the same time, the nanostructures are strongly rooted in the PI surface due to the metallic ions migrating from the inside of the PI film to the surface aggregation, resulting in good interfacial adhesion to the film.

In summary, the fabricated methods of PI composites are very different with respect to diverse demand fields. Considering PI’s unique imide rings and film-forming technology, the researchers can selectively adopt the optimal method according to the application’s needs.

## 4. Application of PI-Based Composites

### 4.1. Thermal Conducting Polymers (LED Lighting and Microelectronics Packaging Technology)

With the rapid development of high-performance wearable microelectronic devices in recent years, the demand for heat-resistant flexible substrates in the industrial and electronic fields has explosively increased. In flexible electronic devices or integrated circuits (IC), developing flexible substrate materials with high thermal conductivity and stability is an inevitable trend, ascribed to the high-temperature process in low-temperature poly-silicon (LTPS) technology [141,142,143]. It is noted that LTPS TFT-based OLED display using a PI substrate usually suffers inferior recoverable residual image characteristics. This is due to the water molecules’ adsorption and/or hydrogen diffusion on the PI surface, leading to a large Von negative shift under the NBTS. When the high-resistance PI material and shielding metal interlayer or ALD–Al_2_O_3_ buffer layer incorporated PI have applied, the recoverable residual image is alleviated and device instability is improved, which simultaneously suppresses moisture and/or hydrogen diffusion from the underlying PI substrates to the channel. The formation of buffer layers with high film density and low surface roughness significantly improves the device characteristics and reliability of TFTs on a PI substrate through better resistance against water molecules.

**Figure 15:**
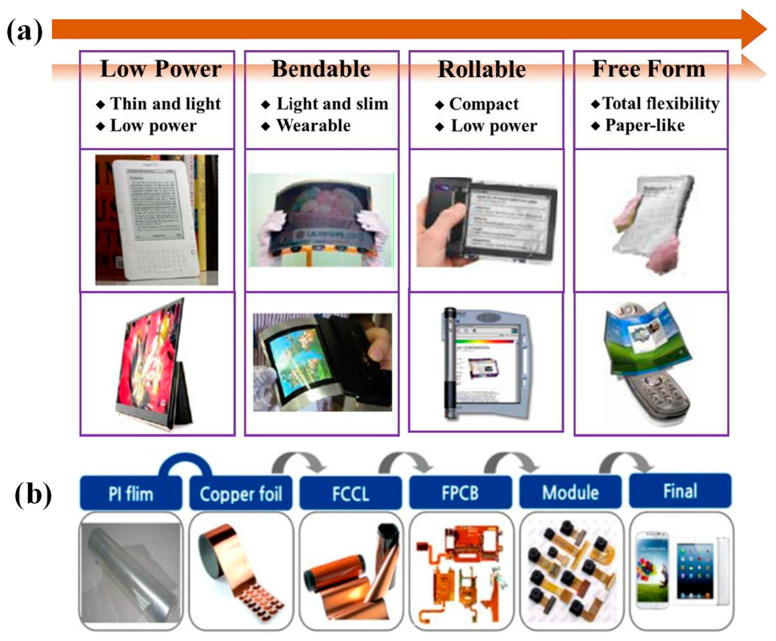
(**a**) Roadmap for flexible products and substrates. (**b**) Flexible printing circuit boards’ (FPCB) industry chains from flexible copper clad laminate (FCCL) to final products. Reprinted with permission from Ref. [151]. 2020, Elsevier.

PI-based materials possessing excellent thermal resistance, good electrical insulation, low dielectric constant, and loss are essential in microelectronic packaging, high-frequency printed circuit boards, flexible display screen, and so on (Figure 15). However, the thermal conductivity of PI itself is in the range of 0.1–0.35 W/mK, which limits its application in the field of heat dissipation. Therefore, some modified fillers are usually introduced, for example, ceramic fillers, such as SiO_2_, aluminum nitride (AlN), and boron nitride (BN) with high thermal conductivity in the range of 100 and 1000 W/mK [144,145,146]; metal fillers, such as Al, Cu, and Ag [147,148]; and carbon-based fillers, such as carbon fiber (CF), CNT, and graphene [149,150]. Combined with functional fillers, PI composites with high thermal conductivity endow their reliability and ductility in communication, automotive, and aerospace applications.

**Figure 16:**
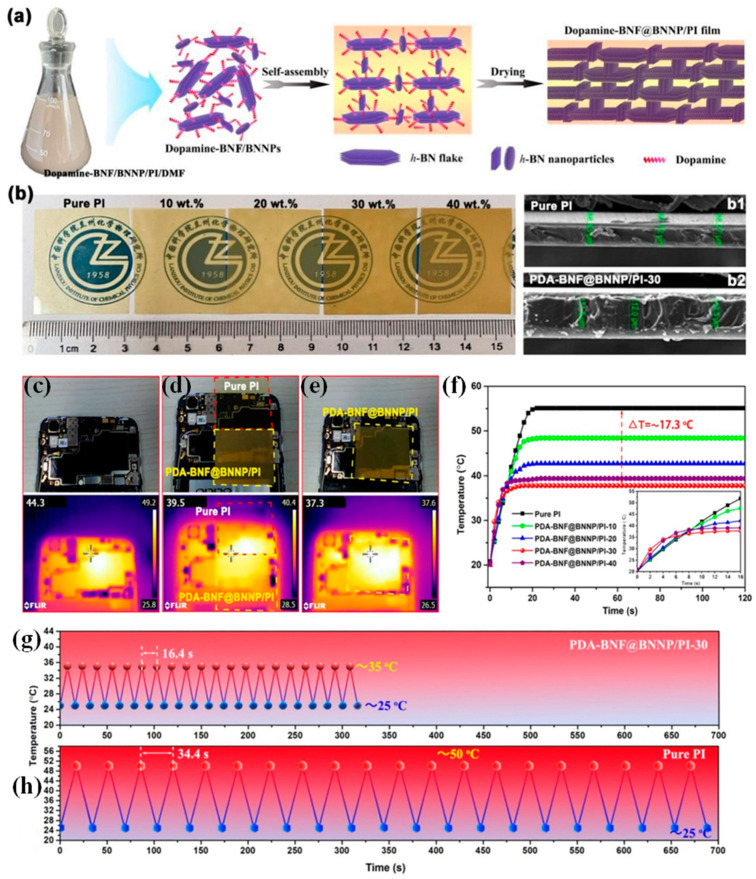
(**a**) A schematic for preparing PDA-BNF@BNNP/PI composite films. (**b**) The transparency of composite films with different contents of PDA-BNF@BNNPs. Including cross-section SEM images of two types films. Thermal infrared images of a running mobile CPU core (**c**) without films, (**d**) covered by composite films, (**e**) covered by PI (up), and covered by composite (down) films. (**f**) Temperature dependence on running time of a mobile CPU core covered by composite films with different contents. Temperature variations of (**g**) PI films and (**h**) composite films during cyclic states. Reprinted with permission from Ref. [152]. 2022, Elsevier.

Many reported works have introduced BN into the PI matrix in different ways to improve its thermal conductivity, such as direct blending, cofilling, and 3D-BN construction. Through the rational assembly of dopamine-modified hexagonal boron nitride flakes and nanoparticles (PDA-BNF@BNNPs), An et al. [152] constructed an orderly layered “brick and plate” structure, which effectively improved the thermal conductivity, thermal stability, and dielectric properties of pure PI. The researchers also used BNNP/PI composites as thin thermal interface materials (TIMs) to explore their heat dissipation performance on the running mobile central processing unit (CPU) cores and observed that the composite film owns higher heat dissipation performance, shorter heating–cooling cycle time, and effectively reduces the stable temperature of the mobile CPU core (Figure 16). This film is a great choice for flexible electronic devices or circuits that require high thermal conductivity and low dielectric losses.

**Figure 17:**
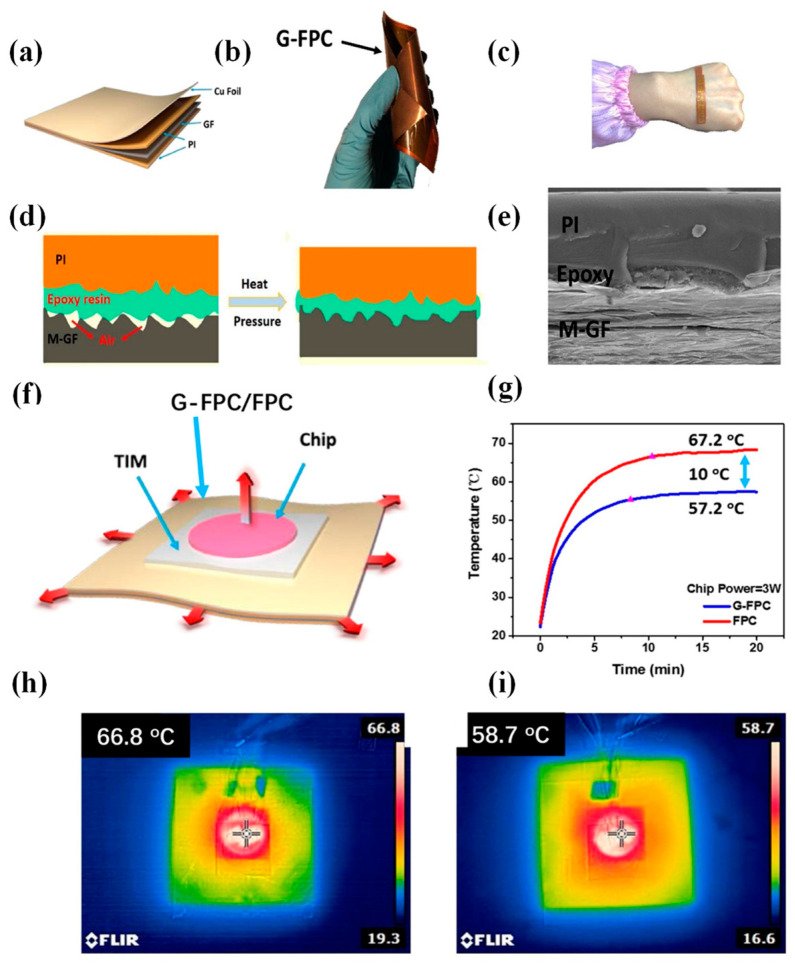
Schematic illustration of G-FPC with (**a**) a sandwich structure and (**b**) flexibility. (**c**) Optical image of a designed flexible print circuit board with M-GF sticking on our hands. (**d**) Schematic of the influence of epoxy resin on contact thermal resistance. (**e**) SEM image of the PI/M-GF boundary is filled with epoxy resin. (**f**) Schematic diagram of the measurement device and heat transfer path for G-FPC and FPC. (**g**) Temperature versus time curve of simulated chips on FPC and G-FPC. Infrared images of (**h**) FPC and (**i**) G-FPC with a chip power of 3 W. Reprinted with permission from Ref. [153]. 2020, Elsevier.

Recently, research on PI–graphene composites with high thermal conductivity for heat dissipation in flexible electronic devices is urgently needed. Wang et al. [153] reported new sandwich flexible printed circuit boards with a multilayer graphene and PI (PI–graphene–PI) structure by laminating and hot-pressing methods, monitoring the surface temperature changes of the analog chips to characterize their heat dissipation capabilities (Figure 17). Benefitting from the good thermal conductivity of the circuit board, the heat generated by the analog chip would quickly travel around the substrate, reducing the die temperature. Compared with unqualified circuit boards, the chip temperature on the PI composite board is reduced by nearly 10 °C, and the thermal conductivity remains at 98% of the original after 10,000 distortions.

Wu et al. [154] reported improving the thermal properties of graphene/PI composite films by in situ polymerization methods. The graphene is grafted to the active site of PAA, and then graphene/PAA is prepared after graphitization. PAAs are converted to PI and inserted on graphene sheets. The inserted PI acts as a “solder” to join the graphene sheet into a large piece during high-temperature annealing. This method can effectively improve the thermal conductivity of the polymer, further fabricated in flexible electronic devices or circuits.

Other works reported that Ag, Al, Cu, and other metallic fillers are dispersed into the PI matrix, which can effectively improve the thermal conductivity of polymer composites, but it destroys the electrical insulation performance to a certain extent. Therefore, this composite can only be applied in fields with low electrical insulation requirements.

### 4.2. Gas Separation Membranes

Membrane-based gas separation has significant advantages, such as no phase change in separation operation, low energy cost, and simple equipment demand [155,156,157]. For feasible gas separation processes, high selectivity and high permeability are the basic requirements of membranes. The permeability, diffusion, and selectivity of gases such as He, O_2_, N_2_, CH_4_, and CO_2_ are generally evaluated. PI materials exhibit extremely high gas selectivity and excellent thermal, mechanical, and chemical stability, rendering them one of the most suitable membrane materials for gas separation [158,159]. This chemical structure of PI-based materials for gas separation membranes should satisfy the following aspects: (1) the backbone can inhibit the flow in the segments of the chain, which should be the rigid chain structure; (2) prevent polymer chain accumulation; and (3) weaken the interaction between the chains. In 1989, Japan Ube developed the first commercial PI-based industrial gas separation membrane to separate H_2_. Then in 1994, the company developed a PI-based gas separation membrane capable of separating CO_2_/CH_4_.

To further improve the performance of the separation membranes, inorganic nanomaterials are often introduced into the PI matrix. By synergizing the processing versatility of PI with the separation properties of inorganic molecular sieves to form organic–inorganic hybrid materials, the high permeability and selectivity of composite membranes can be achieved, and the presence of inorganic phases can also limit the molecular movement of PI chains and lead to an increase in the average distance between chains. The research of PI-SiO_2_ and PI-TiO_2_ nanocomposites using tetraethyl orthosilicate (TEOS) and tetrabutyl titanate (TBT) as silicon and titanium sources has been reported by the sol–gel method. Chris [160] and Katsuki [161] prepared a PI–SiO_2_ nanocomposite membrane, which exhibited better gas permeability and selectivity in the presence of high silica compared with pure PI and achieved CO_2_ transmittance an order of magnitude higher than that of PI films with the same thickness. Subsequently, Lua et al. [162] fabricated PI–SiO_2_ composite membranes and studied the gas permeability of He, CO_2_, N_2_, and O_2_, respectively. As a result, the selectivity of He/N_2_, CO_2_/N_2,_ and O_2_/N_2_ was almost twice that of the PI membrane, without obvious permeability enhancement. When the SiO_2_ content was 7.2%, the composite membrane performed the highest selectivities of 11.33 and 319.33 for O_2_/N_2_ and He/N_2_, respectively. In the PI–TiO_2_ nanocomposites prepared by Kong et al., a strong interaction exists between the TiO_2_ phase and the PI matrix. When the TiO_2_ content is 25%, the 0_2_ and H_2_ permeabilities of the nanocomposite membrane were 0.718 and 14.1 barrer, respectively, which were 4.3 times and 3.7 times higher than that of bare PI. As the TiO_2_ content in the composite membrane is higher than 20 wt%, the permeability of the PI/TiO_2_ composite membrane is significantly enhanced, and the selectivity is still maintained at a high level.

Recently, PI-derived carbon molecular sieve membranes (CMS) have also been proposed for gas separation [163,164,165,166]. Carbon molecular sieves can be obtained by pyrolysis of aromatic polymer precursors. The carbonized carbon molecular sieve membrane possesses highly permeable micropores and selective ultrapores, showing a double pore size distribution. A new type of carbon zeolite membrane is derived from the mixture of one polymer (PI) with good thermal stability and another polymer with poor thermal stability, which can obtain higher permeability and mechanical properties by controlled thermal decomposition. This new type of carbon molecular sieve membrane can perfectly replace the traditional gas separation membrane.

### 4.3. Space Applications

In recent decades, with the human exploration of space, it strongly tends to be possible to develop and utilize space resources. Thus, the unique functional integrated materials for aerospace products are constantly being explored. PI materials with various excellent properties have become one focus material for spacecraft components. However, due to the complex space environment, diverse kinds of radiation, atomic oxygen (AO), temperature fluctuations, human activities, and so on would affect the normal work of spacecraft. Therefore, the PI composite materials’ performance urgently needs to be developed to meet all kinds of space environments.

#### 4.3.1. Atomic Oxygen-Resistant Film

**Figure 18:**
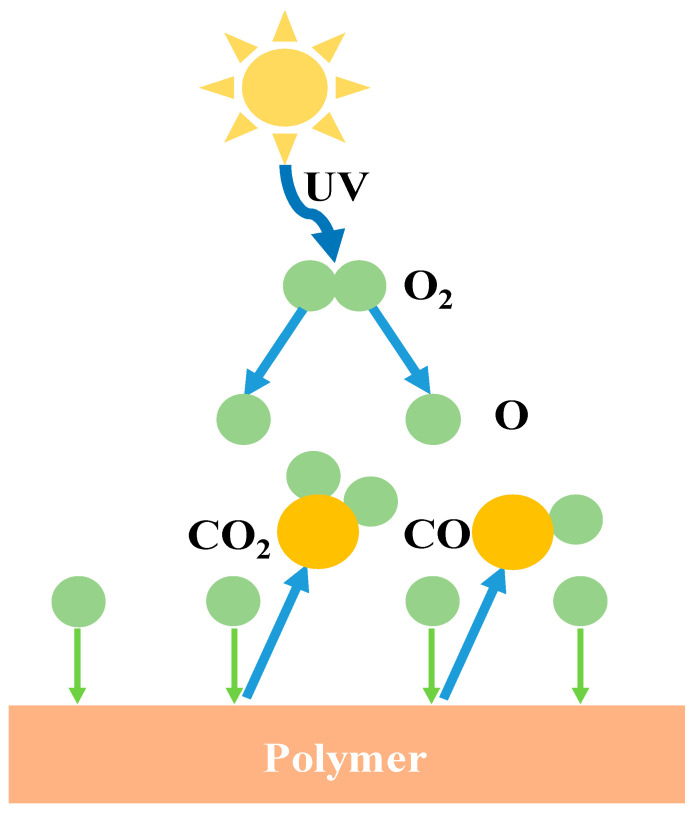
Diagram of atomic oxygen eroding the polymer.

Atomic oxygen is the main gas in the low Earth orbit space [167,168,169]. Many space and ground simulation results have proved that the high energy AO will destroy the carbon chain of the polymer, leading to being oxidized into CO, CO_2_, H_2_O, and other volatile gases, which become the primary damage to PI (Figure 18). One principal engineering method is preparing oxide protective coating on a PI substrate to prevent space erosion.

**Figure 19:**
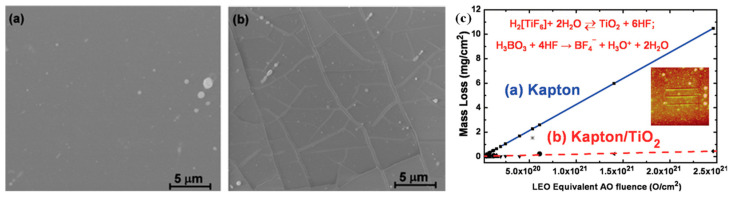
ESEM images of TiO_2_-coated Kapton sample (**a**) before and (**b**) after exposure to 1.5 × 10^19^ atoms cm^−2^ AO fluence. (**c**) Kapton and titania-coated Kapton mass loss as a function of AO fluence. Inset: AFM images of scratch grooves on TiO_2_-coated Kapton. Reprinted with permission from Ref. [174]. 2010, ACS Publications.

Lachance [170] and Russel [171] et al. prepared SiO_2_ and Al_2_O_3_ inorganic oxide coatings on PI surfaces by chemical vapor precipitation, and multilayer film stacks, such as Al_2_O_3_/TiO_2_ and Al_2_O_3_/ZnO, prepared by atomic layer deposition (ALD) can also effectively avoid AO erosion, while ALD owns the disadvantage of high vacuum equipment requirement. Later, the liquid phase deposition method was developed, which generally formed the oxide film by the slow hydrolysis of the aqueous solution of a metal–fluorine complex ([TiF_6_]^2−^) in boric acid and water, achieving a robust interfacial adhesion. Gouzman and Gotlib-Vainshtein et al. [172,173] successfully deposited TiO_2_ and SnO_2_ with a thickness of 100 nm onto a Kapton film by liquid phase deposition, respectively, and simulated an AO exposure environment durability test by an RF plasma of 99.999% pure oxygen gas (usually set an atom value of 10^20^ cm^−2^ order of magnitude as a standard for AO flux measurement in ground simulation facilities), and measured the weight and kinetics of sample erosion, and no chemical composition change has been observed in both structures, antistatic properties, scratch resistance, or thermo-optical properties (Figure 19). In comparison, 2% and 0.3% erosion rates were detected in unprotected films. In contrast, SnO_2_ coatings can provide similar barrier performance to TiO_2_ while significantly reducing electrostatic discharge problems, which has great promise in space material applications.

#### 4.3.2. Shape Memory Materials

With the development of space exploration, shape memory materials (SMM) with light weight, large deformation, and high recovery rate have been applied in deployable structures of wings, satellites, and spacecraft. In addition to the above-mentioned advantages, PIs, as a special material suitable for the space field, have a certain basis of shape memory efficiency [175,176,177,178]. Their large storage modulus difference between the high elastic and glass states endows them with a high shape fixing rate. The π–π conjunction effects and CTC effects provide a high recovery rate. Meanwhile, integrating with functional inorganic particles into PI, such as carbon materials, can availably combine the characteristics of heat, light, and electrodeformation, which will expand SMMs’ application of PI composites in the direction of smart materials.

**Figure 20:**
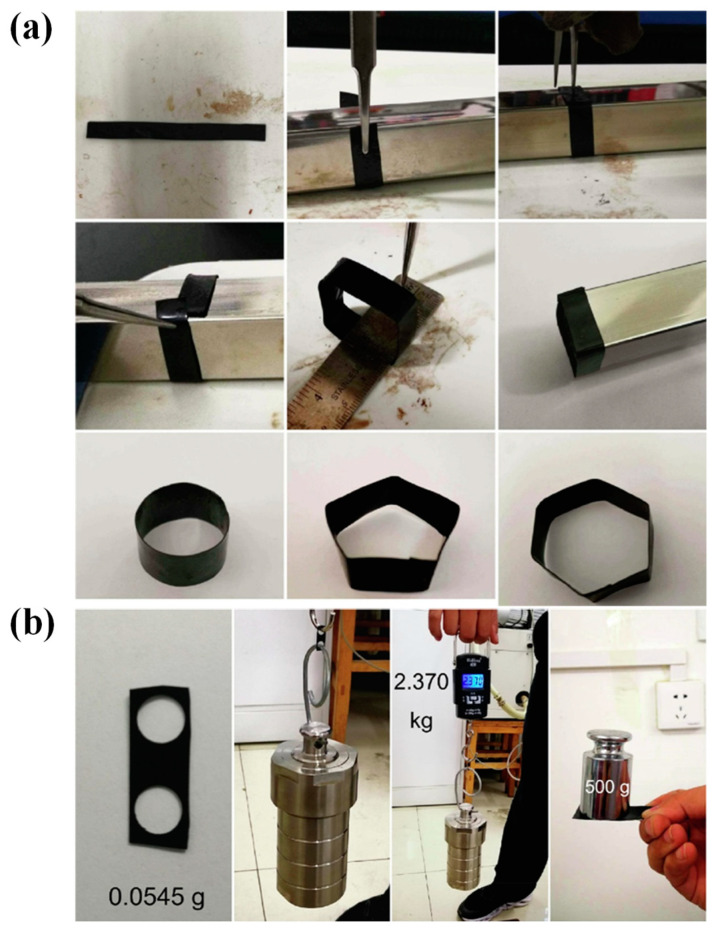
(**a**) Its initial, square, circular, pentagonal, and hexagonal shapes. (**b**) The film with two holes, hanging kettle, and lifting steel counterpoise. Reprinted with permission from Ref. [181]. 2021, Elsevier.

In 2012, Yoonessi [179] et al. introduced graphene into the PI backbone by solution blending. It achieved a series of composite materials, indicating that the introduction of graphene effectively improved the material recovery rate in the shape memory process. It was an early report on shape memory PI. Li et al. [180] incorporated short carbon fibers with a content of 5% into PAA to form an interwoven conductive network, and its T_g_ reached 302 °C, which simultaneously had a high shape recovery stress compared with other electroactive SMMs. Kong et al. [181] integrated short carbon fiber with excellent mechanical properties and carbon black powder with electromagnetic shielding into shape memory PI. These PI composites can be processed into any shape, bearing 2.37 kg of the reaction kettle, still maintaining more than 20 dB shielding effect after 30 shielding cycles (Figure 20). This functional integration material has also become an urgent demand for aerospace products in recent years. The prerequisite is to search for the appropriate combining material and the optimal integration method without destroying the excellent performance of both materials and PI itself. It is still challenging and urgent to figure out in future aerospace projects.

#### 4.3.3. Corona Resistant Material

Corona discharge is a phenomenon that can produce local high temperature and high-energy electron beams and release gases such as ozone (O_3_) and nitric oxide (NO), which is the direct cause of rapid aging and even the breakdown of PI. Due to their unique effect, nanomaterials can balance the electric field inside the polymer and prevent the concentration of the local electric field, thus avoiding partial discharge of the material to improve the corona resistance. It is established that integrating inorganic nanoparticles, such as Al_2_O_3_ and TiO_2_ [182], into PI can significantly improve the conductivity, accelerate the decay rate of space charge, and greatly delay the aging process. At the same time, the ideal thermal conductivity can enhance the heat dissipation of PI, thereby reducing the injection of space charge and weakening the partial discharge effect.

In addition, there are several other frequently used modification additives, such as SiO_2_, Mg(OH)_2_, and ZnO nanoparticles. Compared with pure PIs, the corona life of SiO_2_/PI composites is significantly increased, but with the SiO_2_ content increasing, the elongation at the break of composite films sharply decreased [183,184]. Likewise, the corona resistance and heat resistance of ZnO/PI composites have been strongly improved, but with the increase in ZnO content, their breakdown field strength exhibits different degrees of deterioration, and the volume resistivity also shows a downward trend, which reduces the insulation characteristics of the material to a certain extent [185]. The dielectric coefficient and electrical aging threshold of Mg(OH)_2_/PI composites are increased, but their mechanical strength is slightly weakened compared with pure films [186]. Most current research only focuses on the anticorona performance for regulating PI anticorona modification. However, the anticorona in the space field requires a combination of various aspects, adjusting the properties of PI and improving the uniformity of composite materials. The synergistic modification and improvement of PI antiradiation, corona resistance, antiatomic oxygen, and high thermal conductivity will be the inevitable development trend for future spacecraft’s high-voltage and high-power electrical transmission.

### 4.4. Photocatalytic Application

With the further study of photocatalytic reaction, the design and preparation of supported composite catalysts become the superior method to improve the catalytic activity and selectivity of target products. Inorganic semiconductors, such as TiO_2_, ZnO, and NiO [187,188,189], possess high photocatalytic capability. Still, they can only absorb ultraviolet light, which greatly limits their further application in the photocatalysis field. The band gap of organic semiconductors, including high polymers, such as polyvinyl chloride, carbon nitride, and PI, is relatively narrow, and the light response intensity in the visible range is higher than that of the inorganic. Doping the inorganic into organic semiconductors greatly improves the photocatalytic efficiency in the visible light region. The HOMO and LUMO of PI are located in different structural units, and electron traction exists, which renders a high efficiency of photogenerated carrier separation. PI with high heat and chemical corrosion resistance can be well-matched with metal/inorganic materials. Good tensile strength also facilitates the adhesion of metal/inorganic deposited on the PI surface, achieving excellent catalytic performance. Confining the catalyst on PI substrate has the following advantages: (1) increases the specific surface area and more active sites exposure, and then improves the reaction activity (2) prevents its loss and deactivation in the catalytic process, and facilitates its reusability, enhances the utilization rate of the catalyst. (3) can endure more severe conditions or make into related optical catalytic devices, which broadens the application scope of photocatalysts [190]. Photocatalytic hydrogen production (H_2_), carbon dioxide (CO_2_) reduction, and organic pollutant degradation are the main applications of PI composites.

**Figure 21:**
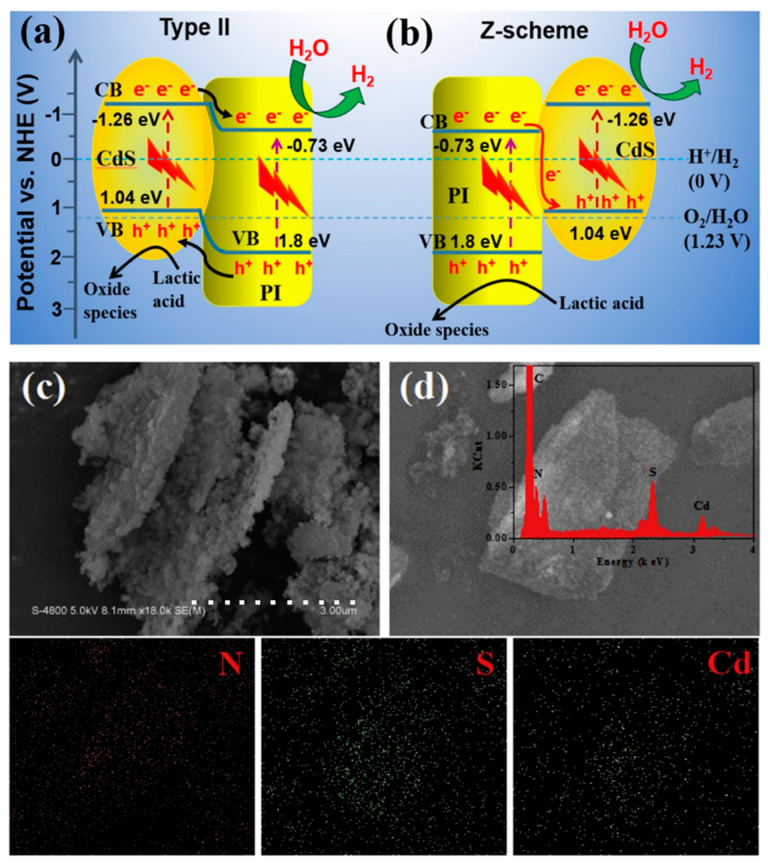
Schematic illustration of (**a**) traditional type-II heterojunction and (**b**) direct Z-scheme charge transfer mechanism: (**c**) 15% CdS/PI, (**d**) SEM–EDX image of 15% CdS/PI, and elemental mapping of the corresponding elements N, S, and Cd. Reprinted with permission from Ref. [194]. 2020, Elsevier.

Ma et al. [191,192] designed and synthesized monolayer MoS_2_ quantum dot/PI (MQDs/PI) and molybdenum trioxide/PI (MoO_3_/PI) composite photocatalysts and found that MoS_2_ and MoO_3_ have strong interaction with PI. The photocatalytic activity is better than Pt/PI in the hydrogen evolution process under the same loading amount. Hu et al. observed that the cadmium sulfide/PI (CdS/PI) composite with Z-type heterojunction achieved high hydrogen production efficiency (Figure 21). The CO_2_ photoreduction rate of a designed Z-type silver chromate/nitrogen-doped graphene/PI nanocomposite is higher than that of most reported works under similar conditions at the same time [193]. Those ascribed to the separation and migration efficiency of photogenerated carriers in this composite material have enhanced in this process. Meanwhile, the recombination rate is also reduced, making the composites have a stronger redox ability, thus enabling excellent catalytic activity in visible light.

**Figure 22:**
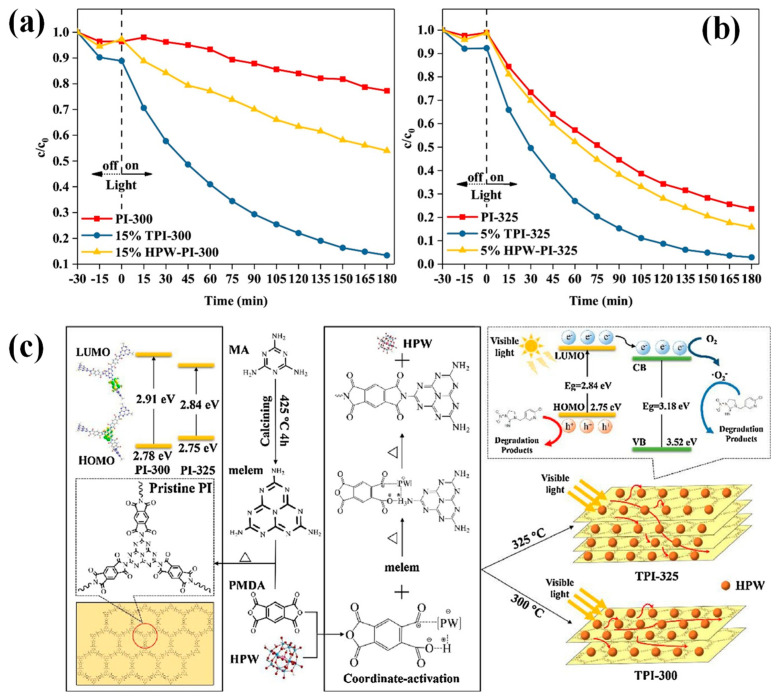
Photocatalytic efficiency of (**a**,**b**) TPI, HPW-PI, and corresponding pristine PI. (**c**) Preparation of TPI composites at different polymerization temperatures and their photocatalytic mechanism under visible light irradiation. Reprinted with permission from Ref. [198]. 2018, Elsevier.

PI-based composites have been extensively studied in the photocatalytic degradation of organic pollutants. Graphene-oxide-coated PI (GO/PI), nano-titanium dioxide/PI/nickel foam (TiO_2_/PI/Ni), silver phosphate/nitrogen-doped graphene/PI (Ag_3_PO_4_/NG/PI), phosphotungstate/PI (HPW/PI) (Figure 22), black molybdenum oxide/PI (MoO_3_/PI), and zinc/PI (ZnS/PI) exhibit excellent degradation efficiency for 2,4-dichlorophenol [195], methylene blue [196], microcystin [197], imidacloprid [198], methyl orange [199], and tetracycline [200], respectively. The heterojunction or coordination bond formed between PI and other compounds will affect the photocatalytic activity of the composite material. In the long run, PI composites demonstrate great development space for applying waste gas and other waste water degradation.

### 4.5. Electrode Applications in Electrocatalysis and Sensing

In electrochemical experiments, the structure and properties of the working electrode directly affect the detection performance. At present, it mainly involves noble metal electrodes, glassy carbon electrodes, and conductive polymer electrodes [201,202,203]. Conductive polymer materials are generally divided into conjugated and composite conductive polymers. Composite conductive polymers comprise nonconductive polymers, such as PI, polyethylene (PE), and conductive fillers, such as carbon and metallic materials. The bond cooperation between the functional groups on the carbon material surface and PI is facilitated by the evenly distributed dopant in the PI matrix, which greatly increases the homogeneity of the electrode and thus keeps the electrochemical signal relatively stable. In addition to the advantages of the carbon electrode, PI/carbon materials have good flexibility, high-temperature resistance, and other characteristics, greatly improving the application range of the materials they load. The modification of functional nanomaterials on PI/carbon electrodes can be effectively used in electrocatalysis, sensing, and detection.

Transition metals and their oxides, alloys, hydroxides, and sulfides perform well in catalytic electrolytic water. Researchers use them to modify PI CNTs, PI graphene oxide, and PI-reduced graphene film electrodes and use them as electrocatalysts for hydrogen evolution (HER) and oxygen evolution reaction (OER). Li et al. [204,205] modified reduced graphene (RGO) sheets, MoO_2_ nanoparticles, and flower-like Co–Ni metals on the surface of PI–CNT films to form electrodes through the coating, deposition, and other methods, respectively. The films show superior catalytic hydrogen and oxygen evolution performance with a low load. Shen et al. [206] electrodeposited the hexagonal CoS–MoS_2_ composite on polyimide/redox graphene (PI/RGO), which has high activity and stability in electrolytic water. This kind of electrocatalyst is made of relatively cheap and abundant elements, which is expected to solve the problem that noble metals are challenging to realize large-scale applications due to their high cost and scarce resources.

Electrochemical sensors based on novel modified electrodes have crucial scientific significance and practical value for analyzing food, drugs, environmental pollutants, and various materials [207,208,209]. Nanometer metal oxides and hydroxides are not only sensitive to light but also sensitive to gas and humidity. They are widely used as sensors after being modified on electrodes. Among them, Ni(OH)_2_ has a good sensing performance for glucose. SnO_2_ is widely used to detect flammable and explosive gases. ZnO is sensitive to pressure, some gases, and water molecules and has piezoelectricity, so it is often used to make varistors, gas, humidity, and so on. Others, such as Co_3_O_4_, NiO, CuO, MnO_2_, Fe_2_O_3_, and AgO, are also very suitable for detecting glucose, hydrogen peroxide, nitrite, and other substances in solution. The researchers prepared PI–CNT–Ni(OH)_2_ [210] and PI–CNT–MoS_X_–Ni(OH)_2_ [211] for the analysis of glucose content in serum. The glucose sensor studied has high sensitivity, low detection limit, stability, and repeatability. In addition, In/RGO/PI/CNT, AuPd/PI/RGO, MoTe_2_/PI/graphene, and Au/PI/graphene are used for the detection of caffeic acid, hydrogen peroxide, hydrazine, sodium nitrite, and other substances.

**Figure 23:**
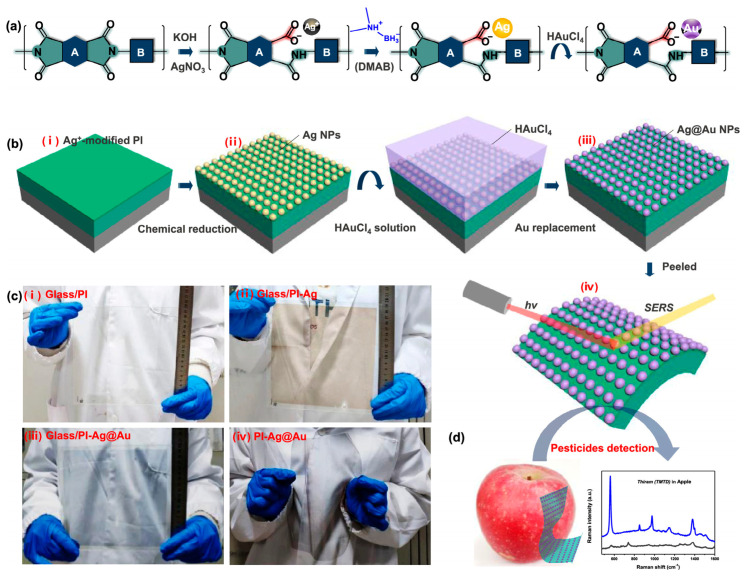
Schematic illustration of (**a**) Ag@Au NP growth on the CPI molecular chain and (**b**) corresponding flexible SERS sensor. (**c**) Photographs of the fabrication process of the SERS sensor. (**d**) Diagram of direct sampling and SERS detection of pesticide on an apple. Reprinted with permission from Ref. [212]. 2018, ACS Publications.

PI-based composite films can be used as reaction substrate electrodes in electrochemistry and have the advantages of wide potential window, wide temperature range, strong acid, alkali corrosion resistance, and so on. Meanwhile, the electrode modified by such nanomaterial realizes rapid and sensitive detection. Coupling with tunable characteristics of the localized surface plasmon resonance (LSPR) in the plasmonic metallic nanostructure, our group introduced a Ag or Ag@Au core–shell nanostructure into the 20 × 20 cm^2^ large-area CPI surface with good homogeneity and robust adhesion by the surface ion exchange method, which applied a flexible surface-enhanced Raman scattering (SERS) sensor with Raman enhancement factor (EF) reaching up to 1.07 × 10^7^ and a low detection limit of 10^−9^ M (Figure 23).

## 5. Conclusions and Prospect

In this review, we summarized the recent studies of PI-based composites for a molecular design, manufacturing process, combination methods, and the relevant applications. By introducing nanofillers including metal, metal alloy, metal oxide, inorganic ceramic, and so on into the PI matrix, combining their respective advantages, the application fields of PI-based materials continue to expand, such as microelectronics, aerospace, and sustainable energy technology.

With the rapid development of technology, the demand for high-performance PIs is increasingly urgent. Improving the versatility of PIs is of great significance and broadens their application prospect. However, the relatively strong hygroscopicity, low thermal conductivity, high surface energy, and dielectric constant are severe obstacles to PI’s widespread use. Future research on PIs should focus on the synthesis, performance improvement, and application of high-performance composites, especially PI–inorganic nanocomposites. PI-based materials with better performance can be prepared by the chemical modification of PIs, formulation of composite materials, and system adjustment to meet the use of high-tech fields.

## Figures and Tables

**Table 1:** Several commercial high-performance optical polymer films worldwide [60].

Company	Country	Product Name	Transmission (%)	Resin	T_g_ (°C)
Mitsubishi Gas Chemical	Japan	Neopulim^®^	89–90	PI	300–489
DuPont Toray	USA	Colorless Kapton^®^	87	PI	>300
Kolon	South Korea	/	89	PI	330–350
Japan Synthetic Rubber	Japan	Lucera^®^	88	/	280
Toyobo	Japan	HM type	91	Polymamideimide (PAI)	225
Nippon Steel Chemical	Japan	Sillplus^®^	91–92	Resin + glass	/
Toray	Japan	Aramid^®^	/	Polyamide (PA)	315
Sumitomo Bakelite	Japan	Sumilite^®^FS-1300	89	Polyethersulfone (PES)	223
Showa Electricity	Japan	Shorayal^®^	92	/	250
Tosoh	Japan	OPS film	93	Polysulfone (PS)	220
Kurabo	Japan	Examid^®^	/	Polyamide (PA)	220

**Table 2:** Typical monomers for CPI synthesis.

Monomers	Chemical Structure	References
1,4-Diaminocyclohexane	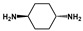	[62]
2,2′-Bis(trifluoromethyl)-4,4′-diaminobiphenyl (TFMB)	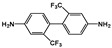	[63]
1,2,4-Cyclohexane tricarboxylic dianhydride (HTA)	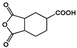	[64]
2,3,5-Tricarboxycyclopentylacetic dianhydride (TCA-AH)	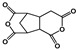	[65]
1,2,3,4-Cyclobutane tetracarboxylic dianhydride (CBDA)	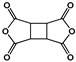	[66]
3,3′,4,4′-Bicyclohexyl tetracarboxylic dianhydride (HBPDA)	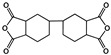	[67]
1R,2S,4S,5R-cyclobutane tetracarboxylic dianhydride (CBDA)	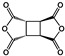	[68]
1S,2R,4S,5R-cyclohexane tetracarboxylic dianhydride (HPMDA)	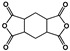	[69]
2,2′-Bis(3,4-dicarboxyphenyl)hexafluoro-propane dianhydride (6FDA)	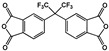	[70]
3,4-Dicarboxy-1,2,3,4-tetrahydro-1-naphthalene succinic dianhydride(TDA)	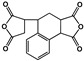	[71]
3,4-Dicarboxy-1,2,3,4-tetrahydro-6-fluoro-1-naphthalene succinicdianhydride (FTDA)	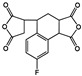	[72]
3,4-Dicarboxy-1,2,3,4-tetrahydro-6-chloro-methyl-1-naphthalenesuccinic dianhydride (CMTDA)	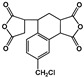	[73]
(5,11-difluoro-5,11-bis (trifluoromethyl)-5,11-dihydro-1 H, 3H-anthraceno [2,3- c: 6, 7-c’] difuran 1,3,7,9-tetraone) (8FDA)	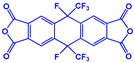	Our work [74]

## Data Availability

All data are contained within this article.

## References

[1] Ohya H., Kudryavsev V.V., Semenova S.I. (1997). Polyimide Membranes: Applications, Fabrications and Properties.

[2] Kash L. (2005). Polyimides and Other High Temperature Polymers: Synthesis, Characterization and Applications.

[3] Yang S.-Y. (2018). Advanced Polyimide Materials: Synthesis, Characterization, and Applications.

[4] Akiyama M., Morofuji Y., Kamohara T., Nishikubo K., Ooishi Y., Tsubai M., Fukuda O., Ueno N. (2007). Preparation of Oriented Aluminum Nitride Thin Films on Polyimide Films and Piezoelectric Response with High Thermal Stability and Flexibility. Adv. Funct. Mater..

[5] Wang Y.-Y., Zhou Z.-H., Zhou C.-G., Sun W.-J., Gao J.-F., Dai K., Yan D.-X., Li Z.-M. (2020). Lightweight and Robust Carbon Nanotube/Polyimide Foam for Efficient and Heat-Resistant Electromagnetic Interference Shielding and Microwave Absorption. ACS Appl. Mater. Interfaces.

[6] Shi Y., Hu A., Wang Z., Li K., Yang S. (2021). Closed-Cell Rigid Polyimide Foams for High-Temperature Applications: The Effect of Structure on Combined Properties. Polymers.

[7] Zheng Z., Liu H., Wu D., Wang X. (2022). Polyimide/Mxene Hybrid Aerogel-Based Phase-Change Composites for Solar-Driven Seawater Desalination. Chem. Eng. J..

[8] Park J.-S., Kim T.-W., Stryakhilev D., Lee J.-S., An S.-G., Pyo Y.-S., Lee D.-B., Mo Y.G., Jin D.-U., Chung H.K. (2009). Flexible Full Color Organic Light-Emitting Diode Display on Polyimide Plastic Substrate Driven by Amorphous Indium Gallium Zinc Oxide Thin-Film Transistors. Appl. Phys. Lett..

[9] Wang B., He B., Wang Z., Qi S., Zhang D., Tian G., Wu D. (2021). Enhanced Impact Properties of Hybrid Composites Reinforced by Carbon Fiber and Polyimide Fiber. Polymers.

[10] He B., Wang B., Wang Z., Qi S., Tian G., Wu D. (2020). Mechanical Properties of Hybrid Composites Reinforced by Carbon Fiber and High-Strength and High-Modulus Polyimide Fiber. Polymer.

[11] Gouzman I., Grossman E., Verker R., Atar N., Bolker A., Eliaz N. (2019). Advances in Polyimide-Based Materials for Space Applications. Adv. Mater..

[12] Sezer Hicyilmaz A., Celik Bedeloglu A. (2021). Applications of Polyimide Coatings: A Review. SN Appl. Sci..

[13] Harasimowicz M., Orluk P., Zakrzewska-Trznadel G., Chmielewski A.G. (2007). Application of Polyimide Membranes for Biogas Purification and Enrichment. J. Hazard. Mater..

[14] Guo Y., Qiu H., Ruan K., Zhang Y., Gu J. (2022). Hierarchically Multifunctional Polyimide Composite Films with Strongly Enhanced Thermal Conductivity. Nanomicro Lett..

[15] Kurosawa T., Higashihara T., Ueda M. (2012). Polyimide Memory: A Pithy Guideline for Future Applications. Polym. Chem..

[16] Ding Y., Hou H., Zhao Y., Zhu Z., Fong H. (2016). Electrospun Polyimide Nanofibers and Their Applications. Prog. Polym. Sci..

[17] Rahman M.M., Nayeem M.A., Nahid S., Bin Alvee S.R., Rashidul Hasan R., Rahman M.A. On-Body Humidity Sensing Antenna with Polyimide for Ban Applications over 5g Networks. Proceedings of the 2020 IEEE International IOT, Electronics and Mechatronics Conference (IEMTRONICS).

[18] Peng J.-J., Qu S.-W., Xia M., Yang S. (2020). Wide-Scanning Conformal Phased Array Antenna for Uav Radar Based on Polyimide Film. IEEE Antennas Wirel. Propag. Lett..

[19] Wang H., Zhu D. (2018). Double Layered Radar Absorbing Structures of Silicon Carbide Fibers/Polyimide Composites. Synth. Met..

[20] Pham H.Q., Kim G., Jung H.M., Song S.W. (2018). Fluorinated Polyimide as a Novel High-Voltage Binder for High-Capacity Cathode of Lithium-Ion Batteries. Adv. Funct. Mater..

[21] Jiang X., Bin Y., Matsuo M. (2005). Electrical and Mechanical Properties of Polyimide–Carbon Nanotubes Composites Fabricated by in Situ Polymerization. Polymer.

[22] Wu G., Cheng Y., Wang Z., Wang K., Feng A. (2017). In Situ Polymerization of Modified Graphene/Polyimide Composite with Improved Mechanical and Thermal Properties. J. Mater. Sci..

[23] Wang J.-Y., Yang S.-Y., Huang Y.-L., Tien H.-W., Chin W.-K., Ma C.-C.M. (2011). Preparation and Properties of Graphene Oxide/Polyimide Composite Films with Low Dielectric Constant and Ultrahigh Strength Via in Situ Polymerization. J. Mater. Chem..

[24] Ahmad Z., Mark J.E. (2001). Polyimide−Ceramic Hybrid Composites by the Sol−Gel Route. Chem. Mater..

[25] Yang C.-P., Su Y.-Y., Hsiao S.-H. (2007). Synthesis and Properties of Low-Color Polyimide/Silica Hybrid Films. J. Appl. Polym. Sci..

[26] Joly C., Goizet S., Schrotter J.C., Sanchez J., Escoubes M. (1997). Sol-Gel Polyimide-Silica Composite Membrane: Gas Transport Properties. J. Membr. Sci..

[27] Jiang H., Xu L., Chen G., Fang X. (2022). Aqueous Solution Blending Route for Preparing Flexible and Antistatic Polyimide/Carbon Nanotube Composite Films with Core-Shell Structured Polyimide/Graphene Microspheres. Polym. Compos..

[28] Shayapat J., Chung O.H., Park J.S. (2015). Electrospun Polyimide-Composite Separator for Lithium-Ion Batteries. Electrochim. Acta.

[29] Zhou X., Ding C., Cheng C., Liu S., Duan G., Xu W., Liu K., Hou H. (2020). Mechanical and Thermal Properties of Electrospun Polyimide/Rgo Composite Nanofibers Via in-Situ Polymerization and in-Situ Thermal Conversion. Eur. Polym. J..

[30] Wu D.C., Shen L., Low J.E., Wong S.Y., Li X., Tjiu W.C., Liu Y., He C.B. (2010). Multi-Walled Carbon Nanotube/Polyimide Composite Film Fabricated through Electrophoretic Deposition. Polymer.

[31] Wang H., Wei M., Zhong Z., Wang Y. (2017). Atomic-Layer-Deposition-Enabled Thin-Film Composite Membranes of Polyimide Supported on Nanoporous Anodized Alumina. J. Membr. Sci..

[32] Ikeda S., Yanagimoto H., Akamatsu K., Nawafune H. (2007). Copper/Polyimide Heterojunctions: Controlling Interfacial Structures through an Additive-Based, All-Wet Chemical Process Using Ion-Doped Precursors. Adv. Funct. Mater..

[33] Ding Q., Miao Y.-E., Liu T. (2013). Morphology and Photocatalytic Property of Hierarchical Polyimide/ZnO Fibers Prepared Via a Direct Ion-Exchange Process. ACS Appl. Mater. Interfaces.

[34] Xing S., Pan Z., Wu X., Chen H., Lv X., Li P., Liu J., Zhai J. (2020). Enhancement of Thermal Stability and Energy Storage Capability of Flexible Ag Nanodot/Polyimide Nanocomposite Films Via in Situ Synthesis. J. Mater. Chem. C.

[35] Jeong S., Song H.C., Lee W.W., Choi Y., Ryu B.-H. (2010). Preparation of Aqueous Ag Ink with Long-Term Dispersion Stability and Its Inkjet Printing for Fabricating Conductive Tracks on a Polyimide Film. J. Appl. Phys..

[36] Oh S.H., Legros M., Kiener D., Gruber P., Dehm G. (2007). In Situ Tem Straining of Single Crystal Au Films on Polyimide: Change of Deformation Mechanisms at the Nanoscale. Acta Mater..

[37] Yadav D., Borpatra Gohain M., Karki S., Ingole P.G. (2022). A Novel Approach for the Development of Low-Cost Polymeric Thin-Film Nanocomposite Membranes for the Biomacromolecule Separation. ACS Omega.

[38] Song J., Ryou M.-H., Son B., Lee J.-N., Lee D.J., Lee Y.M., Choi J.W., Park J.-K. (2012). Co-Polyimide-Coated Polyethylene Separators for Enhanced Thermal Stability of Lithium Ion Batteries. Electrochim. Acta.

[39] Borpatra Gohain M., Karki S., Yadav D., Yadav A., Thakare N.R., Hazarika S., Lee H.K., Ingole P.G. (2022). Development of Antifouling Thin-Film Composite/Nanocomposite Membranes for Removal of Phosphate and Malachite Green Dye. Membranes.

[40] Haight R., White R.C., Silverman B.D., Ho P.S. (1988). Complex Formation and Growth at the Cr– and Cu–Polyimide Interface. J. Vac. Sci. Technol. A.

[41] Muthukrishnan A., Nabae Y., Hayakawa T., Okajima T., Ohsaka T. (2014). Fe-Containing Polyimide-Based High-Performance ORR Catalysts in Acidic Medium: A Kinetic Approach to Study the Durability of Catalysts. Catal. Sci. Technol..

[42] Nam V.B., Shin J., Choi A., Choi H., Ko S.H., Lee D. (2021). High-Temperature, Thin, Flexible and Transparent Ni-Based Heaters Patterned by Laser-Induced Reductive Sintering on Colorless Polyimide. J. Mater. Chem. C.

[43] Zhang Y., Ma Z., Ruan K., Gu J. (2022). Multifunctional Ti_3_C_2_T_x_-(Fe_3_O_4_/Polyimide) Composite Films with Janus Structure for Outstanding Electromagnetic Interference Shielding and Superior Visual Thermal Management. Nano Res..

[44] Yoda S., Hasegawa A., Suda H., Uchimaru Y., Haraya K., Tsuji T., Otake K. (2004). Preparation of a Platinum and Palladium/Polyimide Nanocomposite Film as a Precursor of Metal-Doped Carbon Molecular Sieve Membrane Via Supercritical Impregnation. Chem. Mater..

[45] Nasreen S., Baczkowski M.L., Treich G.M., Tefferi M., Anastasia C., Ramprasad R., Cao Y., Sotzing G.A. (2019). Sn-Polyester/Polyimide Hybrid Flexible Free-Standing Film as a Tunable Dielectric Material. Macromol. Rapid Commun..

[46] Ishii J., Yokotsuka H., Saito T., Hasegawa M. (2011). Strategy for Improvement of Non-Flammability in Functional Polyimides. J. Photopolym. Sci. Technol..

[47] Sava I., Asandulesa M., Zocher K., Kruth A., Kolb J.F., Bodnar W., Witte K., Ishizaki T., Miron C. (2019). Electrical and Mechanical Properties of Polyimide Films Treated by Plasma Formed in Water and Isopropanol. React. Funct. Polym..

[48] Zhang P., Zhang K., Dou S., Zhao J., Yan X., Li Y. (2020). Mechanical, Dielectric, and Thermal Attributes of Polyimides Stemmed out of 4, 4′-Diaminodiphenyl Ether. Crystals.

[49] Liaw D.-J., Wang K.-L., Huang Y.-C., Lee K.-R., Lai J.-Y., Ha C.-S. (2012). Advanced Polyimide Materials: Syntheses, Physical Properties and Applications. Prog. Polym. Sci..

[50] Pakhuruddin M.Z., Ibrahim K., Aziz A.A. (2013). Properties of Polyimide Substrate for Applications in Flexible Solar Cells. Optoelectron. Adv. Mater. Rapid Commun..

[51] Sekitani T., Zschieschang U., Klauk H., Someya T. (2010). Flexible Organic Transistors and Circuits with Extreme Bending Stability. Nat. Mater..

[52] Thiel R.C., de Groot H.J.M., de Vries J.W.C. (1984). Use of Kapton Film as a Cryogenic Construction Material. Cryogenics.

[53] Kim J.H., Lan N.X.V., Kulkarni U., Kim C., Cho S.M., Yoo P.J., Kim D., Schroeder M., Yi G.R. (2020). Optically Transparent and Low-CTE Polyethersulfone-Based Nanocomposite Films for Flexible Display. Adv. Mater. Interfaces.

[54] Tan Y.-y., Zhang Y., Jiang G.-l., Zhi X.-x., Xiao X., Wu L., Jia Y.-J., Liu J.-g., Zhang X.-m. (2020). Preparation and Properties of Inherently Black Polyimide Films with Extremely Low Coefficients of Thermal Expansion and Potential Applications for Black Flexible Copper Clad Laminates. Polymers.

[55] You N.-H., Chueh C.-C., Liu C.-L., Ueda M., Chen W.-C. (2009). Synthesis and Memory Device Characteristics of New Sulfur Donor Containing Polyimides. Macromolecules.

[56] Ling Q.-D., Chang F.-C., Song Y., Zhu C.-X., Liaw D.-J., Chan D.S.-H., Kang E.-T., Neoh K.-G. (2006). Synthesis and Dynamic Random Access Memory Behavior of a Functional Polyimide. J. Am. Chem. Soc..

[57] Ke F., Song N., Liang D., Xu H. (2013). A Method to Break Charge Transfer Complex of Polyimide: A Study on Solution Behavior. J. Appl. Polym. Sci..

[58] Huang J.-J., Chen Y.-P., Lien S.-Y., Weng K.-W., Chao C.-H. (2011). High Mechanical and Electrical Reliability of Bottom-Gate Microcrystalline Silicon Thin Film Transistors on Polyimide Substrate. Curr. Appl. Phys..

[59] Yang S.-Y., Yuan L.-L., Yang S.-Y. (2018). Advanced Polyimide Films. Advanced Polyimide Materials: Synthesis, Characterization, and Applications.

[60] Liu J.-g., Ni H.-j., Wang Z.-h., Yang S.-y., Zhou W.-f. (2015). Colorless and Transparent High-Temperature-Resistant Polymer Optical Films–Current Status and Potential Applications in Optoelectronic Fabrications. Optoelectronics—Materials and Devices.

[61] Ni H.-j., Liu J.-g., Wang Z.-h., Yang S.-y. (2015). A Review on Colorless and Optically Transparent Polyimide Films: Chemistry, Process and Engineering Applications. J. Ind. Eng. Chem..

[62] Jin Q., Yamashita T., Horie K., Yokota R., Mita I. (1993). Polyimides with Alicyclic Diamines. I. Syntheses and Thermal Properties. J. Polym. Sci. A Polym. Chem..

[63] Hasegawa M., Fujii M., Ishii J., Yamaguchi S., Takezawa E., Kagayama T., Ishikawa A. (2014). Colorless Polyimides Derived from 1s,2s,4r,5r-Cyclohexanetetracarboxylic Dianhydride, Self-Orientation Behavior During Solution Casting, and Their Optoelectronic Applications. Polymer.

[64] Hasegawa M., Kasamatsu K., Koseki K. (2012). Colorless Poly(Ester Imide)S Derived from Hydrogenated Trimellitic Anhydride. Eur. Polym. J..

[65] Banerjee S., Madhra M.K., Salunke A.K., Jaiswal D.K. (2003). Synthesis and Properties of Fluorinated Polyimides. 3. Derived from Novel 1,3-Bis[3′-Trifluoromethyl-4′(4″-Amino Benzoxy) Benzyl] Benzene and 4,4-Bis[3′-Trifluoromethyl-4′(4-Amino Benzoxy) Benzyl] Biphenyl. Polymer.

[66] Suzuki H., Abe T., Takaishi K., Narita M., Hamada F. (2000). The Synthesis and X-Ray Structure of 1,2,3,4-Cyclobutane Tetracarboxylic Dianhydride and the Preparation of a New Type of Polyimide Showing Excellent Transparency and Heat Resistance. J. Polym. Sci. A.

[67] Kaneya Y., Arakawa Y., Suzuki K. (2014). Polyimide Precursor Composition, Polyimide Film, and Transparent Flexible Film. Google Patent.

[68] Uchida A., Hasegawa M., Takezawa E., Yamaguchi S., Ishikawa A., Kagayama T. (2012). (1r*,2s*,4s*,5r*)-Cyclohexane-1,2:4, 5-Tetracarboxylic Dianhydride. Acta Crystallogr. Sect. Sect. E Struct. Rep..

[69] Hasegawa M., Hirano D., Fujii M., Haga M., Takezawa E., Yamaguchi S., Ishikawa A., Kagayama T. (2013). Solution-Processable Colorless Polyimides Derived from Hydrogenated Pyromellitic Dianhydride with Controlled Steric Structure. J. Polym. Sci. A.

[70] Li T.-L., Hsu S.L.-C. (2007). Preparation and Properties of a High Temperature, Flexible and Colorless Ito Coated Polyimide Substrate. Eur. Polym. J..

[71] Nishikawa M., Matsuki Y., Bessho N., Iimura Y., Kobayashi S. (1995). Characteristics of Polyimide Liquid Crystal Alignment Films for Active Matrix-Lcd Use. J. Photopolym. Sci. Technol..

[72] Zhang X.-M., Song Y.-Z., Liu J.-G., Yang S.-Y. (2016). Synthesis and Properties of Cost-Effective Light-Color and Highly Transparent Polyimide Films from Fluorine-Containing Tetralin Dianhydride and Aromatic Diamines. J. Photopolym. Sci. Technol..

[73] Guo Y.-z., Song H.-w., Zhai L., Liu J.-g., Yang S. (2012). Synthesis and Characterization of Novel Semi-Alicyclic Polyimides from Methyl-Substituted Tetralin Dianhydride and Aromatic Diamines. Polym. J..

[74] Li F., Liu J., Liu X., Wang Y., Gao X., Meng X., Tu G. (2018). High Performance Soluble Polyimides from Ladder-Type Fluorinated Dianhydride with Polymorphism. Polymers.

[75] Acar O., Varis S., Isık T., Tirkes S., Demir M.M. (2018). Synthesis and Characterization of Novel High Temperature Structural Adhesives Based on Nadic End Capped Mda-Btda-Oda Copolyimide. Mater. Res. Express.

[76] Ni H., Xing Y., Dai X., Zhang D., Li J., Liu J., Yang S., Chen X. (2020). Intrinsically Heat-Sealable Polyimide Films with Atomic Oxygen Resistance: Synthesis and Characterization. High Perform. Polym..

[77] Chen S., Hu P., Greiner A., Cheng C., Cheng H., Chen F., Hou H. (2008). Electrospun Nanofiber Belts Made from High Performance Copolyimide. Nanotechnology.

[78] Yu X., Liang W., Cao J., Wu D. (2017). Mixed Rigid and Flexible Component Design for High-Performance Polyimide Films. Polymers.

[79] Sheng S.-R., Zhang W., Lu C., Wan J., Liu X.-L., Song C.-S. (2012). Synthesis and Characterization of New Cardo Poly(Ether Imide)S Derived from 9,9-Bis [4-(4-Aminophenoxy)Phenyl]Xanthene. J. Appl. Polym. Sci..

[80] Zhang X.-L., Song C., Wei M.-H., Huang Z.-Z., Sheng S.-R. (2019). Organosoluble and Transparent Cardo Polyimides with High T_g_ Derived from 9,9-Bis(4-Aminophenyl)Xanthene. High Perform. Polym..

[81] Tapaswi P.K., Ha C.S. (2019). Recent Trends on Transparent Colorless Polyimides with Balanced Thermal and Optical Properties: Design and Synthesis. Macromol. Chem. Phys..

[82] Hasegawa M., Takahashi S., Tsukuda S., Hirai T., Ishii J., Yamashina Y., Kawamura Y. (2019). Symmetric and Asymmetric Spiro-Type Colorless Poly(Ester Imide)S with Low Coefficients of Thermal Expansion, High Glass Transition Temperatures, and Excellent Solution-Processability. Polymer.

[83] Yu X.-H., Liu J.-N., Wu D.-Y. (2019). Colorless PI Structure Design and Evaluation for Achieving Low Cte Target. Mater. Today Commun..

[84] Hasegawa M. (2017). Development of Solution-Processable, Optically Transparent Polyimides with Ultra-Low Linear Coefficients of Thermal Expansion. Polymers.

[85] Chen Y.-C., Su Y.-Y., Hsiao F.-Z. (2020). The Synthesis and Characterization of Fluorinated Polyimides Derived from 2′-Methyl-1,4-Bis-(4-Amino-2-Trifluoromethylphenoxy)Benzene and Various Aromatic Dianhydrides. J. Macromol. Sci. A.

[86] DeMeuse M.T. (2011). Biaxial Stretching of Film: Principles and Applications.

[87] Meador M.A.B., Malow E.J., Silva R., Wright S., Quade D., Vivod S.L., Guo H., Guo J., Cakmak M. (2012). Mechanically Strong, Flexible Polyimide Aerogels Cross-Linked with Aromatic Triamine. ACS Appl. Mater. Interfaces.

[88] Al-Enizi A.M., Zagho M.M., Elzatahry A.A. (2018). Polymer-Based Electrospun Nanofibers for Biomedical Applications. Nanomaterials.

[89] Kadavil H., Zagho M., Elzatahry A., Altahtamouni T. (2019). Sputtering of Electrospun Polymer-Based Nanofibers for Biomedical Applications: A Perspective. Nanomaterials.

[90] Zhang M., Song W., Tang Y., Xu X., Huang Y., Yu D. (2022). Polymer-Based Nanofiber–Nanoparticle Hybrids and Their Medical Applications. Polymers.

[91] Wen X., Xiong J., Lei S., Wang L., Qin X. (2021). Diameter Refinement of Electrospun Nanofibers: From Mechanism, Strategies to Applications. Adv. Fiber Mater..

[92] Baheti V., Mishra R., Militky J., Behera B.K. (2014). Influence of Noncellulosic Contents on Nano Scale Refinement of Waste Jute Fibers for Reinforcement in Polylactic Acid Films. Fibers Polym..

[93] Zhang X., Dong J., Pan D., Yang G., Su F., Ji Y., Liu C., Shen C. (2021). Constructing Dual Thermal Conductive Networks in Electrospun Polyimide Membranes with Highly Thermally Conductivity but Electrical Insulation Properties. Adv. Compos. Hybrid Mater..

[94] Rogalski J.J., Zhang H., Yao J., Bastiaansen C.W.M., Peijs T. (2020). High-Modulus Rotary Jet Spun Co-Polyimide Nanofibers and Their Composites. Nanocomposites.

[95] Jeong S.-H., Kim J.-K., Lim Y.-W., Hwang H.-B., Kwon H.-Y., Bae B.-S., Jin J. (2018). Squid Pen-Inspired Chitinous Functional Materials: Hierarchical Chitin Fibers by Centrifugal Jet-Spinning and Transparent Chitin Fiber-Reinforced Composite. APL Mater..

[96] Hatami M. (2018). Production of Polyimide Ceria Nanocomposites by Development of Molecular Hook Technology in Nano-Sonochemistry. Ultrason. Sonochem..

[97] Li J., Song G., Yu J., Wang Y., Zhu J., Hu Z. (2017). Preparation of Solution Blown Polyamic Acid Nanofibers and Their Imidization into Polyimide Nanofiber Mats. Nanomaterials.

[98] Yu L., Zhang H., Yu F., Liu Y., Jia L., Zhao W., Li P., Wang H., Zhu P., Li B. (2022). Blow Spinning of Polyimide/SiO_2_ Composite Fibrous Sponges with Excellent Adsorption Capacity and Recyclability. ACS Appl. Polym. Mater..

[99] Zhang M., Niu H., Wu D. (2018). Polyimide Fibers with High Strength and High Modulus: Preparation, Structures, Properties, and Applications. Macromol. Rapid. Commun..

[100] Monsef K., Homayoonfal M., Davar F. (2020). Engineering Arrangement of Nanoparticles within Nanocomposite Membranes Matrix: A Suggested Way to Enhance Water Flux. Polym.-Plast. Technol. Mater..

[101] Niu H., Qi S., Han E., Tian G., Wang X., Wu D. (2012). Fabrication of High-Performance Copolyimide Fibers from 3, 3′, 4, 4′-Biphenyltetracarboxylic Dianhydride, P-Phenylenediamine and 2-(4-Aminophenyl)-6-Amino-4 (3h)-Quinazolinone. Mater. Lett..

[102] Xu W., Ding Y., Jiang S., Ye W., Liao X., Hou H. (2016). High Permittivity Nanocomposites Fabricated from Electrospun Polyimide/BaTiO_3_ Hybrid Nanofibers. Polym. Compos..

[103] Reneker D.H., Chun I. (1996). Nanometre Diameter Fibres of Polymer, Produced by Electrospinning. Nanotechnology.

[104] Chen Y., Han D., Ouyang W., Chen S., Hou H., Zhao Y., Fong H. (2012). Fabrication and Evaluation of Polyamide 6 Composites with Electrospun Polyimide Nanofibers as Skeletal Framework. Compos. B Eng..

[105] Huang C., Chen S., Reneker D.H., Lai C., Hou H. (2006). High-Strength Mats from Electrospun Poly(P-Phenylene Biphenyltetracarboximide) Nanofibers. Adv. Mater..

[106] Miao Y.-E., Zhu G.-N., Hou H., Xia Y.-Y., Liu T. (2013). Electrospun Polyimide Nanofiber-Based Nonwoven Separators for Lithium-Ion Batteries. J. Power Sources.

[107] Chen S., Han D., Hou H. (2011). High Strength Electrospun Fibers. Polym. Adv. Technol..

[108] Huang C., Wang S., Zhang H., Li T., Chen S., Lai C., Hou H. (2006). High Strength Electrospun Polymer Nanofibers Made from Bpda–Pda Polyimide. Eur. Polym. J..

[109] Guo Y., Lyu Z., Yang X., Lu Y., Ruan K., Wu Y., Kong J., Gu J. (2019). Enhanced Thermal Conductivities and Decreased Thermal Resistances of Functionalized Boron Nitride/Polyimide Composites. Compos. B Eng..

[110] Liu X., Ji T., Li N., Liu Y., Yin J., Su B., Zhao J., Li Y., Mo G., Wu Z. (2019). Preparation of Polyimide Composites Reinforced with Oxygen Doped Boron Nitride Nano-Sheet as Multifunctional Materials. Mater. Des..

[111] Li Y., Lv L., Huang W., Zhu Y., Long F., Zheng W., Qu Q., Zheng H. (2022). In Situ Polymerized and Imidized Si@Polyimide Microcapsules with Flexible Solid-Electrolyte Interphase and Enhanced Electrochemical Activity for Li-Storage. ChemElectroChem.

[112] Zhu J., Lim J., Lee C.-H., Joh H.-I., Kim H.C., Park B., You N.-H., Lee S. (2014). Multifunctional Polyimide/Graphene Oxide Composites Via in Situ Polymerization. J. Appl. Polym. Sci..

[113] Guo Y., Xu G., Yang X., Ruan K., Ma T., Zhang Q., Gu J., Wu Y., Liu H., Guo Z. (2018). Significantly Enhanced and Precisely Modeled Thermal Conductivity in Polyimide Nanocomposites with Chemically Modified Graphene Via in Situ Polymerization and Electrospinning-Hot Press Technology. J. Mater. Chem. C.

[114] Chen B., Li X., Li X., Jia Y., Yang J., Yang G., Li C. (2017). Friction and Wear Properties of Polyimide-Based Composites with a Multiscale Carbon Fiber-Carbon Nanotube Hybrid. Tribol. Lett..

[115] Kwon J., Kim J., Lee J., Han P., Park D., Han H. (2014). Fabrication of Polyimide Composite Films Based on Carbon Black for High-Temperature Resistance. Polym. Compos..

[116] Shen J., Li F., Cao Z., Barat D., Tu G. (2017). Light Scattering in Nanoparticle Doped Transparent Polyimide Substrates. ACS Appl. Mater. Interfaces.

[117] Huang J.-W., Wen Y.-L., Kang C.-C., Yeh M.-Y. (2007). Preparation of Polyimide-Silica Nanocomposites from Nanoscale Colloidal Silica. Polym. J..

[118] Liu J., Tian G., Qi S., Wu Z., Wu D. (2014). Enhanced Dielectric Permittivity of a Flexible Three-Phase Polyimide–Graphene–BaTiO_3_ Composite Material. Mater. Lett..

[119] Pei J.-Y., Zha J.-W., Zhou W.-Y., Wang S.-J., Zhong S.-L., Yin L.-J., Zheng M.-S., Cai H.-W., Dang Z.-M. (2019). Enhancement of Breakdown Strength of Multilayer Polymer Film through Electric Field Redistribution and Defect Modification. Appl. Phys. Lett..

[120] Liu W.-D., Zhu B.-K., Zhang J., Xu Y.-Y. (2007). Preparation and Dielectric Properties of Polyimide/Silica Nanocomposite Films Prepared from Sol-Gel and Blending Process. Polym. Adv. Technol..

[121] Zhang K., Ma Z., Fu Q., Deng H. (2022). Multi-Layered Boron Nitride/Polyimide High-Temperature Capacitor Dielectric Film. Mater. Today Energy.

[122] Liu X.-J., Zheng M.-S., Chen G., Dang Z.-M., Zha J.-W. (2022). High-Temperature Polyimide Dielectric Materials for Energy Storage: Theory, Design, Preparation and Properties. Energy Environ. Sci..

[123] Hsiao Y.-S., Chang-Jian C.-W., Huang T.-Y., Chen Y.-L., Huang C.-W., Huang J.-H., Wu N.-J., Hsu S.-C., Chen C.-P. (2023). Lightweight Flexible Polyimide-Derived Laser-Induced Graphenes for High-Performance Thermal Management Applications. Chem. Eng. J..

[124] Park J., Kim W., Aggawal Y., Shin K., Choi E.H., Park B. (2022). Highly Efficient and Stable Organic Light-Emitting Diodes with Inner Passivating Hole-Transfer Interlayers of Poly(Amic Acid)-Polyimide Copolymer. Adv. Sci..

[125] Wang Y., Wang W., Ding X., Yu D. (2020). Multilayer-Structured Ni-Co-Fe-P/Polyaniline/Polyimide Composite Fabric for Robust Electromagnetic Shielding with Low Reflection Characteristic. Chem. Eng. J..

[126] Ishida A., Sato M. (2008). Ti–Ni–Cu Shape-Memory Alloy Thin Film Formed on Polyimide Substrate. Thin Solid Films.

[127] Liu D., Chi H., Ma C., Song M., Zhang P., Dai P. (2022). Improving in-Plane and out-of-Plane Thermal Conductivity of Polyimide/Boron Nitride Film with Reduced Graphene Oxide by a Moving Magnetic Field Induction. Compos. Sci. Technol..

[128] Strack G., AitElAoud Y., Osgood R.M., Akyurtlu A. (2022). Magnetic Nanoarrays on Flexible Substrates. MRS Adv..

[129] Doan H.N., Tagami S., Vo P.P., Negoro M., Sakai W., Tsutsumi N., Kanamori K., Kinashi K. (2022). Scalable Fabrication of Cross-Linked Porous Centrifugally Spun Polyimide Fibers for Thermal Insulation Application. Eur. Polym. J..

[130] Li J., Zhang X., Ding Y., Zhao S., Ma Z., Zhang H., He X. (2022). Multifunctional Carbon Fiber@Nico/Polyimide Films with Outstanding Electromagnetic Interference Shielding Performance. Chem. Eng. J..

[131] Deng J., Cao D., Yang X., Zhang G. (2022). Cross-Linked Cellulose/Carboxylated Polyimide Nanofiber Separator for Lithium-Ion Battery Application. Chem. Eng. J..

[132] Zheng S., Jiang L., Zhang C., Ma N., Liu X. (2022). Facile and Environment-Friendly Preparation of High-Performance Polyimide Aerogels Using Water as the Only Solvent. Polym. Chem..

[133] Tafreshi O.A., Ghaffari-Mosanenzadeh S., Karamikamkar S., Saadatnia Z., Kiddell S., Park C.B., Naguib H.E. (2022). Novel, Flexible, and Transparent Thin Film Polyimide Aerogels with Enhanced Thermal Insulation and High Service Temperature. J. Mater. Chem. C.

[134] Chen X., Liu H., Zheng Y., Zhai Y., Liu X., Liu C., Mi L., Guo Z., Shen C. (2019). Highly Compressible and Robust Polyimide/Carbon Nanotube Composite Aerogel for High-Performance Wearable Pressure Sensor. ACS Appl. Mater. Interfaces.

[135] Lin J., Liu Y., Yang W., Xie Z., Zhang P., Li X., Lin H., Chen G., Lei Q. (2014). Structure and Mechanical Properties of the Hybrid Films of Well Dispersed SiO_2_ Nanoparticle in Polyimide (PI/SiO_2_) Prepared by Sol–Gel Process. J. Polym. Res..

[136] Lei Y., Huo J., Liao H. (2018). Fabrication and Catalytic Mechanism Study of CeO_2_-Fe_2_O_3_-ZnO Mixed Oxides on Double Surfaces of Polyimide Substrate Using Ion-Exchange Technique. Mater. Sci. Semicond. Process..

[137] Luo M., Liu Z., Wang Q., Liu R., Xu Y., Wang K., Shi X., Ye S. (2022). Surface Engineering on Polyimide–Silver Films in Low-Cost, Flexible Humidity Sensors. ACS Appl. Mater. Interfaces.

[138] Wang Y., Ding J., Li N., Ding L., Li D. (2017). Conductive Silver Coatings with Ultra-Low Silver Consumption on Polyimide Film: Via a Mild Surface Ion Exchange Self-Metallization Method. J. Mater. Chem. C.

[139] Pan C., Chen S.-J., Huang Y.-H., Wang L., Luo J.-L., Fu X.-Z. (2022). A Facile Method to Fabricate Lightweight Copper Coated Polyimide Film Current Collectors for Lithium-Ion Batteries. J. Power Sources.

[140] Chen L., Liang B., Lv J., Chen M., Hu J., Zeng K., Yang G. (2022). Route to a Porous Carbon Nanofiber Membrane Containing Fexcy/Fe by Facile in Situ Ion-Exchange Functionalization of the Paa Carboxyl Group: Exemplified by a Supercapacitor. ACS Appl. Energy Mater..

[141] Tu H.-Y., Chang T.-C., Tsao Y.-C., Tai M.-C., Zheng Y.-Z., Tu Y.-F., Kuo C.-W., Wu C.-C., Tsai Y.-L., Tsai T.-M. (2022). Abnormal Two-Stage Degradation on P-Type Low-Temperature Polycrystalline-Silicon Thin-Film Transistor under Hot Carrier Conditions. IEEE Electron Device Lett..

[142] Jin Y., Ao W., Liu B., Lu Y., Li L., Xi W., Jiang J., Ma M., Hu F., Fu D. (2022). Systematic Investigation on Anode Etching Residue Widely Generated in Manufacturing of Low-Temperature Polycrystalline-Si Active Matrix Organic Light-Emitting Diode. J. Soc. Inf. Disp..

[143] Ji D., Li T., Hu W., Fuchs H. (2019). Recent Progress in Aromatic Polyimide Dielectrics for Organic Electronic Devices and Circuits. Adv. Mater..

[144] Xiao H., Huang Z.X., Zhang Z.P., Rong M.Z., Zhang M.Q. (2021). Highly Thermally Conductive Flexible Copper Clad Laminates Based on Sea-Island Structured Boron Nitride/Polyimide Composites. Compos. Sci. Technol..

[145] Ding D., Zou M., Wang X., Qin G., Zhang S., Chan S.Y., Meng Q., Liu Z., Zhang Q., Chen Y. (2022). Thermal Conductivity of Polydisperse Hexagonal Bn/Polyimide Composites: Iterative Emt Model and Machine Learning Based on First Principles Investigation. Chem. Eng. J..

[146] Feng C.-P., Wei F., Sun K.-Y., Wang Y., Lan H.-B., Shang H.-J., Ding F.-Z., Bai L., Yang J., Yang W. (2022). Emerging Flexible Thermally Conductive Films: Mechanism, Fabrication, Application. Nano-Micro Lett..

[147] Zhao Z.-B., Liu J.-D., Du X.-Y., Wang Z.-Y., Zhang C., Ming S.-F. (2022). Fabrication of Silver Nanoparticles/Copper Nanoparticles Jointly Decorated Nitride Flakes to Improve the Thermal Conductivity of Polymer Composites. Colloids Surf. A Physicochem. Eng. Asp..

[148] Zhou H., Guo K., Ma S., Wang C., Fan X., Jia T., Zhang Z., Xu H., Xing H., Wang D. (2022). A Triple-Layer Structure Flexible Sensor Based on Nano-Sintered Silver for Power Electronics with High Temperature Resistance and High Thermal Conductivity. Chem. Eng. J..

[149] Weng M., Luo X., Jian L., Liang J., Hu J., Liu Y., Zhang J., Feng X., Min Y. (2022). Lutidine Catalyzed Highly Thermal Conductive Graphite Polyimide Films Via Controlling Grain Size. Appl. Surf. Sci..

[150] Li S., Zheng Z., Liu S., Chi Z., Chen X., Zhang Y., Xu J. (2022). Ultrahigh Thermal and Electric Conductive Graphite Films Prepared by G-C3n4 Catalyzed Graphitization of Polyimide Films. Chem. Eng. J..

[151] Yi C., Li W., Shi S., He K., Ma P., Chen M., Yang C. (2020). High-Temperature-Resistant and Colorless Polyimide: Preparations, Properties, and Applications. Sol. Energy.

[152] An L., Yang Z., Zeng X., Hu W., Yu Y., Zhang J., Wang Q. (2022). Flexible and Quasi-Isotropically Thermoconductive Polyimide Films by Guided Assembly of Boron Nitride Nanoplate/Boron Nitride Flakes for Microelectronic Application. Chem. Eng. J..

[153] Wang Y., Wang H., Liu F., Wu X., Xu J., Cui H., Wu Y., Xue R., Tian C., Zheng B. (2020). Flexible Printed Circuit Board Based on Graphene/Polyimide Composites with Excellent Thermal Conductivity and Sandwich Structure. Compos. Part A Appl. Sci. Manuf..

[154] Wu X., Li H., Cheng K., Qiu H., Yang J. (2019). Modified Graphene/Polyimide Composite Films with Strongly Enhanced Thermal Conductivity. Nanoscale.

[155] Kong J., Liu J., Jia P., Qi N., Chen Z., Xu S., Li N. (2022). Synergistic Effect of Thermal Crosslinking and Thermal Rearrangement on Free Volume and Gas Separation Properties of 6fda Based Polyimide Membranes Studied by Positron Annihilation. J. Membr. Sci..

[156] Wang C., Cai Z., Xie W., Jiao Y., Liu L., Gong L., Zhang Q.-W., Ma X., Zhang H., Luo S. (2022). Finely Tuning the Microporosity in Dual Thermally Crosslinked Polyimide Membranes for Plasticization Resistance Gas Separations. J. Membr. Sci..

[157] Shi Y., Wang Z., Shi Y., Zhu S., Zhang Y., Jin J. (2022). Synergistic Design of Enhanced Π–Π Interaction and Decarboxylation Cross-Linking of Polyimide Membranes for Natural Gas Separation. Macromolecules.

[158] Sanaeepur H., Ebadi Amooghin A., Bandehali S., Moghadassi A., Matsuura T., Van der Bruggen B. (2019). Polyimides in Membrane Gas Separation: Monomer’s Molecular Design and Structural Engineering. Prog. Polym. Sci..

[159] Tong X., Wang S., Dai J., Wang S., Zhao X., Wang D., Chen C. (2022). Synthesis and Gas Separation Properties of Aromatic Polyimides Containing Noncoplanar Rigid Sites. ACS Appl. Polym. Mater..

[160] Cornelius C.J., Marand E. (2002). Hybrid Silica-Polyimide Composite Membranes: Gas Transport Properties. J. Membr. Sci..

[161] Kusakabe K., Ichiki K., Hayashi J.-i., Maeda H., Morooka S. (1996). Preparation and Characterization of Silica—Polyimide Composite Membranes Coated on Porous Tubes for CO_2_ Separation. J. Membr. Sci..

[162] Lua A.C., Shen Y. (2013). Preparation and Characterization of Polyimide–Silica Composite Membranes and Their Derived Carbon–Silica Composite Membranes for Gas Separation. Chem. Eng. J..

[163] Hou M., Li L., Song J., Xu R., He Z., Lu Y., Pan Z., Song C., Wang T. (2022). Polyimide-Derived Carbon Molecular Sieve Membranes for High-Efficient Hydrogen Purification: The Development of a Novel Phthalide-Containing Polyimide Precursor. Sep. Purif. Technol..

[164] Ning X., Koros W.J. (2014). Carbon Molecular Sieve Membranes Derived from Matrimid^®^ Polyimide for Nitrogen/Methane Separation. Carbon.

[165] Wang Z., Ren H., Zhang S., Zhang F., Jin J. (2018). Carbon Molecular Sieve Membranes Derived from Tröger’s Base-Based Microporous Polyimide for Gas Separation. ChemSusChem.

[166] Swaidan R., Ma X., Litwiller E., Pinnau I. (2013). High Pressure Pure- and Mixed-Gas Separation of CO_2_/CH_4_ by Thermally-Rearranged and Carbon Molecular Sieve Membranes Derived from a Polyimide of Intrinsic Microporosity. J. Membr. Sci..

[167] Reddy M.R. (1995). Effect of Low Earth Orbit Atomic Oxygen on Spacecraft Materials. J. Mater. Sci..

[168] Packirisamy S., Schwam D., Litt M.H. (1995). Atomic Oxygen Resistant Coatings for Low Earth Orbit Space Structures. J. Mater. Sci..

[169] Wang R., Dong N., Tian G., Liu G., Zhou B., Qi S., Wu D. (2022). Enhanced Atomic-Oxygen Resistance of Surface-Siliconized Polyimide Film Via an in-Situ Precursor Network at the Interface. Appl. Surf. Sci..

[170] Lachance J., Coïa C., Fozza A.C., Czeremuszkin G., Houdayer A., Wertheimer M.R. (2001). Radiation-Induced Degradation of Polymeric Spacecraft Materials under Protective Oxide Coatings. Nucl. Instrum. Methods Phys. Res..

[171] Cooper R., Upadhyaya H.P., Minton T.K., Berman M.R., Du X., George S.M. (2008). Protection of Polymer from Atomic-Oxygen Erosion Using Al_2_O_3_ Atomic Layer Deposition Coatings. Thin Solid Films.

[172] Gotlib-Vainstein K., Gouzman I., Girshevitz O., Bolker A., Atar N., Grossman E., Sukenik C.N. (2015). Liquid Phase Deposition of a Space-Durable, Antistatic SnO_2_ Coating on Kapton. ACS Appl. Mater. Interfaces.

[173] Gouzman I., Gershevitz O., Grossman E., Eliaz N., Sukenik C.N. (2009). Novel Approach to Space-Survivable Polyimides: Liquid Phase Deposition of Titania Coating on Kapton. AIP Conf. Proc..

[174] Gouzman I., Girshevitz O., Grossman E., Eliaz N., Sukenik C.N. (2010). Thin Film Oxide Barrier Layers: Protection of Kapton from Space Environment by Liquid Phase Deposition of Titanium Oxide. ACS Appl. Mater. Interfaces.

[175] Yang Z., Zhang Y., Li S., Zhang X., Wang T., Wang Q. (2020). Fully Closed-Loop Recyclable Thermosetting Shape Memory Polyimide. ACS Sustain. Chem. Eng..

[176] Xiao X., Kong D., Qiu X., Zhang W., Zhang F., Liu L., Liu Y., Zhang S., Hu Y., Leng J. (2015). Shape-Memory Polymers with Adjustable High Glass Transition Temperatures. Macromolecules.

[177] Narayane D., Taiwade R.V., Sahu K. (2022). Review on Development and Performance of Shape Memory Alloy/Polyimide Thin-Film Composites. Mater. Manuf. Process..

[178] Huang X., Zhang F., Liu Y., Leng J. (2020). Active and Deformable Organic Electronic Devices Based on Conductive Shape Memory Polyimide. ACS Appl. Mater. Interfaces.

[179] Yoonessi M., Shi Y., Scheiman D.A., Lebron-Colon M., Tigelaar D.M., Weiss R.A., Meador M.A. (2012). Graphene Polyimide Nanocomposites; Thermal, Mechanical, and High-Temperature Shape Memory Effects. ACS Nano.

[180] Li X., Wang L., Zhang Z., Kong D., Ao X., Xiao X. (2019). Electroactive High-Temperature Shape Memory Polymers with High Recovery Stress Induced by Ground Carbon Fibers. Macromol. Chem. Phys..

[181] Kong D., Li J., Guo A., Xiao X. (2021). High Temperature Electromagnetic Shielding Shape Memory Polymer Composite. Chem. Eng. J..

[182] Zhao X., Yin J.H., Jin R., Dong J.Y. (2013). Effect of Content and Layer Thickness on the Corona-Resistance of PI/TiO_2_ Nanocomposite Films. Appl. Mech. Mater..

[183] Yao L., Chen L., Chen Y., Sun X. (2016). Effect of SiO_2_ Nanoparticles on the Thermal Properties of Dielectric Composite Films. Int. J. Manuf. Technol. Manag..

[184] Rui J.M. (2013). Preparation of Novel Corona-Resistance Polyimide/Spherical SiO_2_ Hybrid Films. Adv. Mat. Res..

[185] Hu W., Du B., Li J., Liu Y., Liu M. Electrical and Mechanical Characteristics of Polyimide Nanocomposite Films for Wind Generator. Proceedings of the IEEE PES Innovative Smart Grid Technologies.

[186] Wang X., Fan Y., Chen H., Yang R., Zhao W. (2017). Electrical, Mechanical, and Thermal Properties of Mg(OH)_2_/PI Nanocomposite Films. J. Inorg. Organomet. Polym. Mater..

[187] Li J.-Y., Jiang X., Lin L., Zhou J.-J., Xu G.-S., Yuan Y.-P. (2015). Improving the Photocatalytic Performance of Polyimide by Constructing an Inorganic-Organic Hybrid Zno-Polyimide Core–Shell Structure. J. Mol. Catal. A Chem..

[188] Sheng W., Shi J.-L., Hao H., Li X., Lang X. (2020). Polyimide-TiO_2_ Hybrid Photocatalysis: Visible Light-Promoted Selective Aerobic Oxidation of Amines. Chem. Eng. J..

[189] Ramasundaram S., Seid M.G., Lee W., Kim C.U., Kim E.-J., Hong S.W., Choi K.J. (2017). Preparation, Characterization, and Application of TiO_2_-Patterned Polyimide Film as a Photocatalyst for Oxidation of Organic Contaminants. J. Hazard. Mater..

[190] Chu S., Pan Y., Wang Y., Zhang H., Xiao R., Zou Z. (2020). Polyimide-Based Photocatalysts: Rational Design for Energy and Environmental Applications. J. Mater. Chem. A.

[191] Ma C., Zhu H., Zhou J., Cui Z., Liu T., Wang Y., Wang Y., Zou Z. (2017). Confinement Effect of Monolayer MoS_2_ Quantum Dots on Conjugated Polyimide and Promotion of Solar-Driven Photocatalytic Hydrogen Generation. Dalton Trans..

[192] Ma C., Zhou J., Cui Z., Wang Y., Zou Z. (2016). In Situ Growth MoO_3_ Nanoflake on Conjugated Polymer: An Advanced Photocatalyst for Hydrogen Evolution from Water Solution under Solar Light. Sol. Energy Mater. Sol. Cells.

[193] Zhou L., Kamyab H., Surendar A., Maseleno A., Ibatova A.Z., Chelliapan S., Karachi N., Parsaee Z. (2019). Novel Z-Scheme Composite Ag_2_CrO_4_/Ng/Polyimide as High Performance Nano Catalyst for Photoreduction of CO_2_: Design, Fabrication, Characterization and Mechanism. J. Photochem. Photobiol. A.

[194] Hu Y., Hao X., Cui Z., Zhou J., Chu S., Wang Y., Zou Z. (2020). Enhanced Photocarrier Separation in Conjugated Polymer Engineered Cds for Direct Z-Scheme Photocatalytic Hydrogen Evolution. Appl. Catal. B.

[195] Gong Y., Yang B., Zhang H., Zhao X., Zhu C. (2019). Graphene Oxide Enwrapped Polyimide Composites with Efficient Photocatalytic Activity for 2,4-Dichlorophenol Degradation under Visible Light Irradiation. Mater. Res. Bull..

[196] Lei Y., Huo J. (2018). Enhanced Visible-Light Photoelectrochemical and Photoelectrocatalytic Activity of Nano-TiO_2_/Polyimide/Ni Foam Photoanode. Res. Chem. Intermed..

[197] Guo Q., Li H., Zhang Q., Zhang Y. (2018). Fabrication, Characterization and Mechanism of a Novel Z-Scheme Ag_3_PO_4_/Ng/Polyimide Composite Photocatalyst for Microcystin-Lr Degradation. Appl. Catal. B.

[198] Meng P., Heng H., Sun Y., Huang J., Yang J., Liu X. (2018). Positive Effects of Phosphotungstic Acid on the in-Situ Solid-State Polymerization and Visible Light Photocatalytic Activity of Polyimide-Based Photocatalyst. Appl. Catal. B.

[199] Ma C., Zhou J., Zhu H., Yang W., Liu J., Wang Y., Zou Z. (2015). Constructing a High-Efficiency MoO_3_/Polyimide Hybrid Photocatalyst Based on Strong Interfacial Interaction. ACS Appl. Mater. Interfaces.

[200] Li Y., Fu M., Lu P., Hu X., Wang R., Bai J., He Y. (2022). Visible Light Photocatalytic Abatement of Tetracycline over Unique Z-Scheme Zns/PI Composites. Appl. Surf. Sci..

[201] Pumera M., Sánchez S., Ichinose I., Tang J. (2007). Electrochemical Nanobiosensors. Sens. Actuator B Chem..

[202] Sirés I., Brillas E., Oturan M.A., Rodrigo M.A., Panizza M. (2014). Electrochemical Advanced Oxidation Processes: Today and Tomorrow. A Review. Environ. Sci. Pollut. Res. Int..

[203] Wang G., Zhang L., Zhang J. (2012). A Review of Electrode Materials for Electrochemical Supercapacitors. Chem. Soc. Rev..

[204] Li X., Jiang Y., Jia L., Wang C. (2016). MoO_2_ Nanoparticles on Reduced Graphene Oxide/Polyimide-Carbon Nanotube Film as Efficient Hydrogen Evolution Electrocatalyst. J. Power Sources.

[205] Li X., Wang T., Wang C. (2017). An Advanced Flower-Like Co-Ni/PI-CNT Film Electrocatalyst for Oxygen Evolution Reaction. J. Alloys Compd..

[206] Shen X., Xia X., Ye W., Du Y., Wang C. (2017). Hexagram-Like Cos-MoS_2_ Composites with Enhanced Activity for Hydrogen Evolution Reaction. J. Solid State Electrochem..

[207] Kothuru A., Hanumanth Rao C., Puneeth S.B., Salve M., Amreen K., Goel S. (2020). Laser-Induced Flexible Electronics (Life) for Resistive, Capacitive and Electrochemical Sensing Applications. IEEE Sens. J..

[208] Kim H.-U., Kim H.Y., Seok H., Kanade V., Yoo H., Park K.-Y., Lee J.-H., Lee M.-H., Kim T. (2020). Flexible MoS_2_–Polyimide Electrode for Electrochemical Biosensors and Their Applications for the Highly Sensitive Quantification of Endocrine Hormones: Pth, T3, and T4. Anal. Chem..

[209] Huang T.-C., Yeh L.-C., Huang H.-Y., Nian Z.-Y., Yeh Y.-C., Chou Y.-C., Yeh J.-M., Tsai M.-H. (2014). The Use of a Carbon Paste Electrode Mixed with Multiwalled Carbon Nanotube/Electroactive Polyimide Composites as an Electrode for Sensing Ascorbic Acid. Polym. Chem..

[210] Jiang Y., Yu S., Li J., Jia L., Wang C. (2013). Improvement of Sensitive Ni(OH)_2_ Nonenzymatic Glucose Sensor Based on Carbon Nanotube/Polyimide Membrane. Carbon.

[211] Wang Q., Zhang Y., Ye W., Wang C. (2016). Ni(OH)_2_/MoS_X_ Nanocomposite Electrodeposited on a Flexible CNT/PI Membrane as an Electrochemical Glucose Sensor: The Synergistic Effect of Ni(OH)_2_ and MoS_X_. J. Solid State Electrochem..

[212] Liu X., Ma J., Jiang P., Shen J., Wang R., Wang Y., Tu G. (2020). Large-Scale Flexible Surface-Enhanced Raman Scattering (SERS) Sensors with High Stability and Signal Homogeneity. ACS Appl. Mater. Interfaces.

